# Imperfect spoiling in variable flip angle T_1_ mapping at 7T: Quantifying and minimizing impact

**DOI:** 10.1002/mrm.28720

**Published:** 2021-03-01

**Authors:** Nadège Corbin, Martina F. Callaghan

**Affiliations:** ^1^ Wellcome Centre for Human Neuroimaging UCL Queen Square Institute of Neurology University College London London United Kingdom

**Keywords:** 7T, EPG, imperfect spoiling, MPM, T1 mapping, VFA

## Abstract

**Purpose:**

The variable flip angle (VFA) approach to T_1_ mapping assumes perfectly spoiled transverse magnetisation at the end of each repetition time (TR). Despite radiofrequency (RF) and gradient spoiling, this condition is rarely met, leading to erroneous T_1_ estimates ($T_{1}^{\text{app}}$). Theoretical corrections can be applied but make assumptions about tissue properties, for example, a global T_2_ time. Here, we investigate the effect of imperfect spoiling at 7T and the interaction between the RF and gradient spoiling conditions, additionally accounting for diffusion. We provide guidance on the optimal approach to maximise the accuracy of the T_1_ estimate in the context of 3D multi‐echo acquisitions.

**Methods:**

The impact of the spoiling regime was investigated through numerical simulations, phantom and *in* *vivo* experiments.

**Results:**

The predicted dependence of $T_{1}^{\text{app}}$ on tissue properties, system settings, and spoiling conditions was observed in both phantom and *in vivo* experiments. Diffusion effects modulated the dependence of $T_{1}^{\text{app}}$ on both $B_{1}^{+}$ efficiency and T_2_ times.

**Conclusion:**

Error in $T_{1}^{\text{app}}$ can be minimized by using an RF spoiling increment and gradient spoiler moment combination that minimizes T_2_‐dependence and safeguards image quality. Although the diffusion effect was comparatively small at 7T, correction factors accounting for this effect are recommended.

## Abstract 2

Click here for author‐reader discussions

## 1 INTRODUCTION

Quantitative MRI (qMRI) is a powerful tool for investigating human brain microstructure *in vivo*. Key physical properties of the tissue can be quantified by combining weighted images with an appropriate physical model of the MRI signal.^1^ This approach has been used to generate *in vivo* markers of myelin and iron distributions in the brain, see Edwards et al for a review,^2^ and the sensitivity of the longitudinal relaxation time, T_1_, to myelin content^3^ has enabled the *in vivo* investigation of structure‐function relationships.^4^, ^5^, ^6^, ^7^

The variable flip angle (VFA) approach is a time‐efficient method that combines a minimum of two spoiled gradient echo (SPGR) sequences, with different nominal flip angles, to estimate T_1_. Several variants, based on regression (DESPOT_1_ ^8^, ^9^, ^10^, ^11^), numerical minimization,^12^ or using a closed form solution,^13^, ^14^ exist to combine these data. While the approach of Heule et al^12^ provides an analytical solution for the steady‐state SPGR signal that accounts for RF spoiling,^15^ all others assume perfect spoiling, that is, that no transverse magnetisation persists across repetition times (TRs). In practice, gradient and radiofrequency (RF) spoiling are used to improve the validity of this assumption. Nonetheless, it is rarely met, leading to erroneous apparent T_1_ estimates ($T_{1}^{\text{app}}$).

The most commonly used RF spoiling scheme relies on quadratically incrementing the RF pulse phase according to $\phi_{n}=\frac{\phi_{0}}{2}\left(n+1\right)n$ where n is the repetition number and *ϕ*_0_ is the RF spoiling increment,^16^ which has been shown to influence the error in $T_{1}^{\text{app}}$.^17^ In gradient spoiling, voxels are assumed to consist of uniformly distributed isochromats, such that the MR signal sums to zero when the phase distribution imparted across the voxel is an integer multiple of 2π. This spoiling mechanism, and its impact on $T_{1}^{\text{app}}$, is amplified by diffusion effects as previously demonstrated in phantoms using large gradient moments.^18^ However, achieving large moments is demanding on gradient performance and greatly extends the minimum achievable TR, running the risk of negating the benefit of these rapid imaging sequences.

Correction schemes have been proposed to recover the true T_1_ from $T_{1}^{\text{app}}$ at 3T.^17^, ^19^ These use Bloch simulations to model the impact of imperfect spoiling and derive correction factors that depend on the transmit field efficiency $f_{B_{1}^{+}}$. They assume an expected T_2_ and the likely range of T_1_ times and transmit field efficiencies, but to date have neglected diffusion effects.

It is unclear how imperfect spoiling will impact T_1_ estimation at 7T, given T_2_ shortens but T_1_ and $f_{B_{1}^{+}}$ inhomogeneity increase. The sensitivity of 7T is often used to increase resolution. Imparting large spoiler moments across small voxel dimensions is increasingly demanding in terms of time and gradient performance. Considering these points, this study aimed to combine simulations and experiments to answer the following questions at 7T:

1. How do RF and gradient spoiling interact and which combination maximises the accuracy and precision of VFA‐based T_1_ mapping?
2. What impact does the diffusion effect have when considering clinically feasible gradient moments and the impact of the full readout?
3. What are the limitations of applying simulation‐derived correction factors to recover the true T_1_ from $T_{1}^{\text{app}}$?

We address these questions in the context of the multi‐parameter mapping (MPM) protocol, which uses a 3D multi‐echo VFA technique to quantify $T_{1}^{\text{app}}$ and subsequently correct for imperfect spoiling.^17^, ^20^, ^21^ The multi‐echo readout allows the flip angle‐dependent signal intensity at echo time (TE) = 0 ms to be estimated together with $T_{2}^{*}$. The impact of different RF and gradient spoiling combinations on $T_{1}^{\text{app}}$ are investigated as is the robustness of correcting for imperfect spoiling in post‐processing when tissue and sequence parameters vary.

## 2 METHODS

### 2.1 Simulating the sensitivity of $T_{1}^{\text{app}}$ to imperfect spoiling

The SPGR signal was simulated using the EPG formalism (https://sycomore.readthedocs.io/)^22^ incorporating the diffusion‐driven spoiling effect^23^ imparted by the readout and spoiler gradients, applied on the same axis. A net dephasing of *nPi* was simulated per TR and varied from 2*π* to 10*π* with an increment of 2*π*. RF spoiling was simulated with increments *ϕ*_0_ varying from 0° to 179° with an increment of 1°. A wide range of $f_{B_{1}^{+}}$, 40% to 160% with an increment of 30%, was simulated to capture increased transmit field inhomogeneity at 7T. Magnetization transfer (MT) effects were not included in the simulations.

Two SPGR signals, *S*_1_ and *S*_2_, were simulated with a TR of 19.50 ms and flip angles *α*_1_ = 6° and *α*_2_ = 26°, respectively. $T_{1}^{\text{app}}$ was estimated using the exact analytical expression^14^:

(1) $$T_{1}^{\mathrm{app}}=‐\frac{\mathrm{TR}}{\ln\left(E_{1}\right)}\;\text{with}\;E_{1}=\frac{S_{2}‐\frac{S_{1}\;\text{sin}\alpha_{2}^{c}}{\text{sin}\alpha_{1}^{c}}}{S_{2}\cdot \text{cos}\alpha_{2}^{c}‐S_{1}\cdot \frac{\text{cos}\alpha_{1}^{c}\cdot \text{sin}\alpha_{2}^{c}}{\text{sin}\alpha_{1}^{c}}}\;\text{and}\;\left\{\begin{array}{c} \alpha_{1}^{c}=\alpha_{1}.\frac{f_{B_{1}^{+}}}{100}\\ \alpha_{2}^{c}=\alpha_{2}.\frac{f_{B_{1}^{+}}}{100} \end{array}\right)$$

This protocol was simulated for the range of T_1_ and T_2_ times reported for gray matter (GM) and white matter (WM) at 7T: T_1_ varying from 1000 ms to 2000 ms with a step of 250 ms^24^, ^25^, ^26^, ^27^ and T_2_ varying from 35 to 55 ms with an increment of 5 ms.^28^ The diffusion coefficient, D, was varied between 0.6 μm^2^/ms and 1 μm^2^/ms with an interval of 0.1 μm^2^/ms.^29^, ^30^ This protocol was also simulated for the phantom used in this study: T_1_ = 950 ms, T_2_ = 60 and 80 ms, D = 1.7 μm^2^/ms.

The error in $T_{1}^{\text{app}}$ relative to the true T_1_ was computed as: $\varepsilon \left(p\right)=100*\frac{T_{1}^{\text{app}}\left(p\right)‐T_{1}^{\text{true}}}{T_{1}^{\text{true}}}$ with *p* a vector of simulation parameters, that is, $p=\left[T_{1}^{\mathrm{true}},T_{2},D,nPi,\phi ,f_{B_{1}^{+}}\right]$.

The SD of the error with all but one parameter fixed is used as a proxy to evaluate the sensitivity of $T_{1}^{\text{app}}$ to that parameter. For example, the sensitivity to T_2_ was computed as follows:

(2) $$Sensitivity_{T_{2}}\left(T_{1}^{\mathrm{true}},D,nPi,\phi ,f_{B_{1}^{+}}\right)=\sqrt{\frac{1}{N_{T2}}\sum_{\text{All}T_{2}}{\left(\varepsilon \left(p\right)‐\bar{\varepsilon }\left(p\right)\right)}^{2}}$$

Where $\bar{\varepsilon }\left(p\right)=\frac{1}{N_{T2}}\sum_{\text{All}T_{2}}\varepsilon \left(p\right)$ and *N_T_*_2_ the number of T_2_ times simulated.

### 2.2 Estimating correction parameters for imperfect spoiling

For a given set of parameters (T_2_, D, *ϕ*_0_ and nPi), correction factors were estimated as described in Ref. 17:

1. For every $f_{B_{1}^{+}}$, coefficients $A\left(f_{B_{1}^{+}}\right)$ and $B\left(f_{B_{1}^{+}}\right)$ were estimated by linear regression:

(3) $$T_{1}=A\left(f_{B_{1}^{+}}\right)+B\left(f_{B_{1}^{+}}\right).T_{1}^{\mathrm{app}}$$

- A second degree polynomial was fitted to the coefficients A and B:

$$\left\{\begin{array}{c} A=a_{2}f_{B_{1}^{+}}^{2}+a_{1}f_{B_{1}^{+}}+a_{0}\\ B=b_{2}f_{B_{1}^{+}}^{2}+b_{1}f_{B_{1}^{+}}+b_{0} \end{array}\right)$$

A set of coefficients $\left(a_{i},b_{i},i\in \left[0,1,2\right]\right)$ was computed for every pair $\left(\phi_{0},\;nPi\right)$ based on simulations with different T_2_ and D combinations applicable to both *in vivo* and phantom experiments (Table 1A).

**TABLE 1:** A, Parameters used to determine the correction factors for phantom and *in vivo* acquisitions. B, Data acquired in each of the 7T imaging sessions

A. Correction factors								
Set	$f_{B_{1}^{+}}$ [%]	In vivo			Phantom			
		T_1_ [ms]	T_2_ [ms]	D [µm^2^/ms]	T_1_ [ms]	T_2_ [ms]	D [µm^2^/ms]	
1	40:30:160	1000:150:2000	35	0.8	650:150:1250	60	1.7	
2	40:30:160	1000:150:2000	45	0.8	650:150:1250	80	1.7	
3	40:30:160	1000:150:2000	55	0.8	650:150:1250	60	0	
4	40:30:160	1000:150:2000	35	0				
5	40:30:160	1000:150:2000	55	0	*(min:step:max)*			

B. Acquisitions								
In vivo	Sequence	FA [°]	Φ_0_ [°]	nPi [π]	Voltage	Sampling	Acquisition time [min]	Phantom
Session 1,2,3,4	SPGR	6	50,117,120, 144	2	×1	elliptical	5.01	✓
	SPGR	26	50,117,120, 144	2	×1	elliptical	5.01	✓
	SPGR	6	50,117,120, 144	6	×1	elliptical	5.01	✓
	SPGR	26	50,117,120, 144	6	×1	elliptical	5.01	✓
	BSS				×1	elliptical	3.52	✓
	SPGR	6	50,117,120, 144	2	×1.6	elliptical	5.01	✘
	SPGR	26	50,117,120, 144	2	×1.6	elliptical	5.01	✘
	SPGR	6	50,117,120, 144	6	×1.6	elliptical	5.01	✘
	SPGR	26	50,117,120, 144	6	×1.6	elliptical	5.01	✘
	BSS		50,117,120, 144		×1.6	elliptical	3.52	✘
Session 5	SPGR	26	117	6	×1	elliptical	5.01	✘
Session 6	SPGR	26	50	2	×1	full	5.42	✘
	SPGR	26	50	6	×1	full	5.42	✘
	SPGR	26	117	2	×1	full	5.42	✘
	SPGR	26	117	6	×1	full	5.42	✘
Session 7	SE‐EPI	1 slice, 13 TEs, variable $B_{1}^{+}$ region					13	✓
	SE‐EPI	1 slice, 13 TEs, homogeneous $B_{1}^{+}$ region					13	✘
	IR‐SE‐EPI	1 slice, 10 TIs, variable $B_{1}^{+}$ region					10	✓
	IR‐SE‐EPI	1 slice, 10 TIs, homogeneous $B_{1}^{+}$ region					10	✘
	DW‐SE‐EPI	32 slices , 3 b‐values					0.5	✓

BSS = Bloch‐Siegert Shift based $B_{1}^{+}$ mapping.

### 2.3 Image artifact due to imperfect spoiling

Gradient spoiling assumes uniformly distributed isochromats within a voxel, which is violated by partial voluming with different (or no) isochromats.^31^ Bloch simulations, neglecting diffusion, were used to simulate a 1D grid of 100 isochromats with T_1_ = 1500 ms and T_2_ = 45 ms. Sequence parameters matching the *in vivo* acquisitions, described later, were adopted with *ϕ*_0_ = 50° or 117°. An off‐resonance frequency of 1 kHz was attributed to one quarter of the spins, mimicking partial voluming of fat and water at 7T. The simulated signal was computed, for each TR, as the integral of the transverse magnetisation. The signal phase was plotted as a function of both phase‐encoding directions, labeled partitions (inner loop, 120 acquired) and lines (outer loop, 192 acquired).

### 2.4 Acquisitions

All data were acquired on a Siemens 7T Terra using a head coil with 8 transmit and 32 receive channels (Nova Medical).

#### 2.4.1 Reference measurements

Reference T_1_ and T_2_ maps were obtained from single‐slice spin‐echo echo‐planar‐imaging (EPI) acquisitions with (IR‐SE‐EPI) and without (SE‐EPI) inversion preparation, respectively. Key parameters were: TR = 10 s, in‐plane field of view (FOV) of 192 × 192 mm^2^ with 1.2 × 1.2 mm^2^ in‐plane resolution, slice thickness of 3.5 mm, acceleration factor of 3 and partial Fourier (6/8). To estimate T_2_, 13 acquisitions were obtained with variable TE (29, 34, 39, 44, 49, 59, 69, 79, 89, 99, 109, 119, or 129 ms). To estimate T_1_, data were acquired with 10 inversion times (100, 170, 200, 280, 470, 780, 1300, 2100, 3600, or 5000 ms) with a fixed TE of 29 ms.

The apparent diffusion coefficient (ADC) was measured with diffusion‐weighted spin‐echo EPI acquisitions. Key parameters were: 34 axial slices, 1.4 mm isotropic resolution, TE/TR = 63/3700 ms, multiband factor 2, in‐plane acceleration factor 2, fat saturation preparation. Three acquisitions with diffusion encoding along x, y or z, were obtained with b‐values of 1000 s/mm^2^, 700 s/mm^2^ or 0 s/mm^2^. T_2_ and ADC were estimated with a mono‐exponentional decay using a log‐linear fit and T_1_ was estimated with a nonlinear least‐squares fit to the inversion recovery signal equation.

#### 2.4.2 MPM protocols

The SPGR data were acquired with an in‐house sequence at 1 mm isotropic resolution over a FOV of 192 × 192 × 160 mm^3^ using settings that matched the simulations. Flip angles of $\alpha_{1}=6^{\circ }$ (referred to as “PDw”) and $\alpha_{2}=26^{\circ }$ (“T_1_w”) were achieved with rectangular excitation pulses of duration 80 µs and 1500 µs, respectively, to match the pulse power $\left(B_{1}^{2}\times \text{pulse}\;\text{duration}\right)$. Six echoes were acquired with TE ranging from 2.56 ms to 11.66 ms in steps of 1.82 ms using a TR of 19.5 ms. The spoiler gradient moment was varied across protocols by changing its duration. Elliptical sampling and partial Fourier (6/8) in both phase‐encoding directions were used to achieve tolerable session durations. Elliptical sampling was only turned off for Session 6 (c.f. Table 1B).

$f_{B_{1}^{+}}$ was mapped using an in‐house sequence exploiting the Bloch‐Siegert shift^32^ and reconstructed in real‐time with in‐house code implemented in Gadgetron.^33^ Relevant parameters were: single echo, TE/TR = 6.77/40 ms, 14° flip angle, FOV of 256 × 256 × 192 mm^3^ with 4 mm isotropic resolution. The $B_{1}^{+}$‐encoding was achieved with a Fermi pulse of duration 2 ms, 2 kHz off‐resonance frequency and 190° flip angle. An RF spoiling increment of 90° was used as required for the interleaved acquisition scheme with short TR adopted here.^34^

#### 2.4.3 Imaging sessions

To evaluate the effects in a simplified scenario, a phantom was constructed of 1.5% (w/v) agarose in a 1 mM copper sulphate solution. Reference T_1_, T_2_, and ADC measurements were acquired, along with T_1_ mapping data using the eight MPM protocols (4$\Phi_{0}$ × 2 *nPi*).

To evaluate the effects *in vivo*, reference and MPM data were acquired in a healthy volunteer (female, 40 y), with approval from the local ethics committee. Each MPM protocol was repeated with the transmitter’s reference voltage increased by 60% to test the simulation‐based hypothesis that the impact of the T_2_ used in the imperfect spoiling correction would increase at higher $f_{B_{1}^{+}}$. Additional scanning sessions were performed to facilitate co‐registration to an independent data set and to explore the impact of k‐space sampling on image artifacts. The acquisitions are summarised in Table 1B.

#### 2.4.4 T_1_ estimation

A modified version of the hMRI toolbox (hMRI.info)^20^ was used to process each PDw/T_1_w pair acquired under the same conditions, that is, consistent *nPi*, *ϕ*_0_ and reference voltage. The PDw and T_1_w signals at TE = 0 were estimated from the log‐linear fit of the echoes from both contrasts^35^ and used to estimate $T_{1}^{\text{app}}$, using Equation (1) and correcting for $f_{B_{1}^{+}}$ with the corresponding $B_{1}^{+}$ map. A total of 8 $T_{1}^{\text{app}}$ maps were computed for the phantom (2 *nPi* × 4*ϕ*_0_) and 16 for the *in vivo* case (2 *nPi* × 2 transmitter voltages × 4*ϕ*_0_).

Corrected phantom T_1_ maps were constructed for each of the eight acquisition conditions by applying three sets (two T_2_ values with diffusion, and one without) of simulation‐based imperfect spoiling correction parameters calculated for the corresponding combination of *ϕ*_0_ and *nPi* resulting in 24 corrected T_1_ maps. Corrected *in vivo* T_1_ maps were also constructed for each condition by applying five sets of correction factors (three T_2_ values with diffusion, and two without) resulting in 80 corrected T_1_ maps.

### 2.5 Analysis

*In vivo*, one acquisition per session (*T_1_w*, *echo 1*, *nPi* = *6π*, *nominal voltage)* was segmented using SPM12.4.^36^ GM and WM masks were defined by those voxels for which the probability of belonging to the given tissue class exceeded 0.9. Global GM and WM masks were computed from the intersection of these session‐specific masks.

To ensure equivalent processing, particularly spatial interpolation, each T_1_ and $B_{1}^{+}$ map was co‐registered to the T_1_‐weighted image acquired independently in Session 5. Separately, the 3D T_1_ maps were co‐registered to the single‐slice reference maps with SPM12.6, which supports 2D input as reference for 3D volumes.

#### 2.5.1 Phantom

To investigate the dependence of $T_{1}^{\text{app}}$ on $f_{B_{1}^{+}}$, voxels were partitioned into 5% $f_{B_{1}^{+}}$ intervals. The median $T_{1}^{\text{app}}$ was computed per bin, plotted against $f_{B_{1}^{+}}$ and compared to simulations using T_2_ = 80 ms, D = 1.7 µm^2^/ms and T_1_ = 950 ms. The same analysis was performed on the corrected T_1_ maps.

#### 2.5.2 In vivo

The dependence of $T_{1}^{\text{app}}$ on $f_{B_{1}^{+}}$ was investigated for each MPM protocol, with the nominal transmitter reference voltage. To minimize confounding anatomical variability, the analysis was restricted to voxels for which the reference T_1_ time was between 1100 ms and 1350 ms in the slice with greatest transmit field inhomogeneity (Supporting Information Figure S1, which is available online). Voxels were partitioned into 2% $f_{B_{1}^{+}}$ intervals. The results were compared to simulations using the mean reference values (ie, T_2_ = 45 ms, D = 0.8 µm^2^/ms and T_1_ = 1250 ms).

To investigate the dependence of $T_{1}^{\text{app}}$ on T_2,_ the reference T_2_ map was used to partition voxels into 2 ms intervals across the T_2_ range. To minimize uncontrolled $f_{B_{1}^{+}}$ variance, the reference slice with least transmit field inhomogeneity was used (Supporting Information Figure S1). $f_{B_{1}^{+}}$ dependence was investigated by analysing both the nominal and high (x1.6) transmitter voltage $T_{1}^{\text{app}}$ maps. The analysis was again restricted to voxels with reference T_1_ between 1100 and 1350 ms to minimize anatomical variability. Median $T_{1}^{\text{app}}$ per bin was computed for each imaging scenario (*nPi*, *ϕ*_0_ and transmitter voltage), plotted against T_2_ and compared to simulations with D = 0.8 µm^2^/ms, T_1_ = 1250 ms and $f_{B_{1}^{+}}$ = 70 and 130%.

For each *in vivo* T_1_ map, from each of the MPM sessions, the distribution of $T_{1}^{\text{app}}$ and corrected T_1_ times within GM and WM were plotted for voxels with $f_{B_{1}^{+}}$ between 95% and 105% according to the nominal voltage $f_{B_{1}^{+}}$ map.

## 3 RESULTS

### 3.1 Simulated error in $T_{1}^{\text{app}}$: dependence on $B_{1}^{+}$ efficiency and tissue properties

**FIGURE 1:**
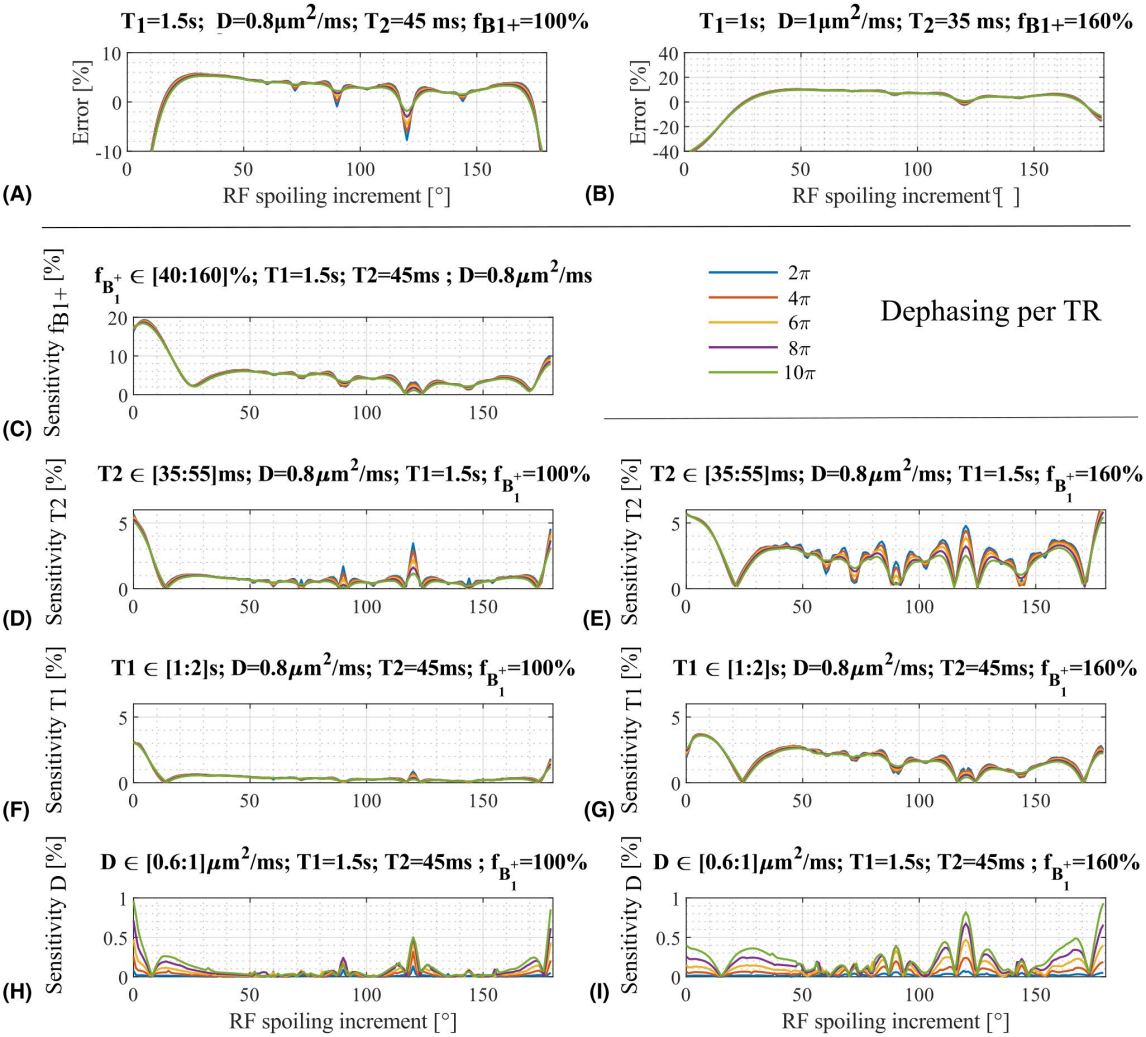
Numerical simulations, for each spoiling condition, of $T_{1}^{\text{app}}$ error (ε) in two specific cases: T_1_ = 1.5 s, D = 0.8 µm^2^/ms, T_2_ = 45 ms, and $f_{B_{1}^{+}}$ = 100% (A); T_1_ = 1 s, D = 1 µm^2^/ms, T_2_ = 35 ms, and $f_{B_{1}^{+}}$ = 160% (B). Sensitivity of $T_{1}^{\text{app}}$ to $B_{1}^{+}$ efficiency (C), the true T_2_ time (D‐E), the true T_1_ time (F‐G), and the diffusion coefficient (H‐I). The sensitivity to T_1_, T_2_, and D are computed in two conditions: $B_{1}^{+}$ efficiency of 100% (D‐F‐H) or 160% (E‐G‐I)

With parameters in the middle of their simulated ranges (Figure 1A), $T_{1}^{\text{app}}$ matched the true T_1_ when *ϕ*_0_ was approximately 15°, 89°, 91°, 117°, 123°, and 174° when *nPi* = 2π. Slightly different *ϕ*_0_ were required if *nPi* differed. Over‐estimation of T_1_ peaked at 6% with *ϕ*_0_ = 30° and *nPi* = 2π.

Higher $f_{B_{1}^{+}}$ and D, coupled with shorter T_2_ and T_1_, required markedly different *ϕ*_0_ (26°, 118°, 123°, and 171°) for $T_{1}^{\text{app}}$ to match the true T_1_ (Figure 1B), although dependence on *nPi* was reduced. However, the error was larger with over‐estimation peaking at 10% with *ϕ*_0_ = 48°.

Overall, no *ϕ*_0_ and *nPi* combination provided accurate T_1_ estimates for all tissue properties and $B_{1}^{+}$ efficiencies. The impact of gradient spoiling on the error was variable across the different conditions simulated.

$T_{1}^{\text{app}}$ was most sensitive to $f_{B_{1}^{+}}$ (Figure 1C). The degree of sensitivity was highly dependent on *ϕ*_0_. It reached 6% for the commonly used value of 50°, but fell below 2% for 117° when T_2_, T_1_, and D were fixed to 45 ms, 1500 ms, and 0.8 µm^2^/ms, respectively.

Sensitivity to T_2_ (and T_1_) was markedly lower falling below 2% (and 1%) for most *ϕ*_0_ when $f_{B_{1}^{+}}$ was optimal (ie, 100%, Figure 1D,F) but increased to 4% (and 3%) when $f_{B_{1}^{+}}$ was 160% (Figure 1E,G).

$T_{1}^{\text{app}}$ was least sensitive to D over the range of values tested. With T_1_ and T_2_ of 1500 ms and 45 ms, respectively, the maximum sensitivity did not exceed 1%, even for high $f_{B_{1}^{+}}$ (Figure 1H,I). Higher *nPi* increased sensitivity to D.

For some *ϕ*_0_, the sensitivity to T_2_, T_1_ and $f_{B_{1}^{+}}$ decreased as *nPi* increased (eg, 110° and 85°), whereas for others the opposite was true (eg, 60° and 72°).

### 3.2 Simulated error in T_1_: impact of correction parameters

**FIGURE 2:**
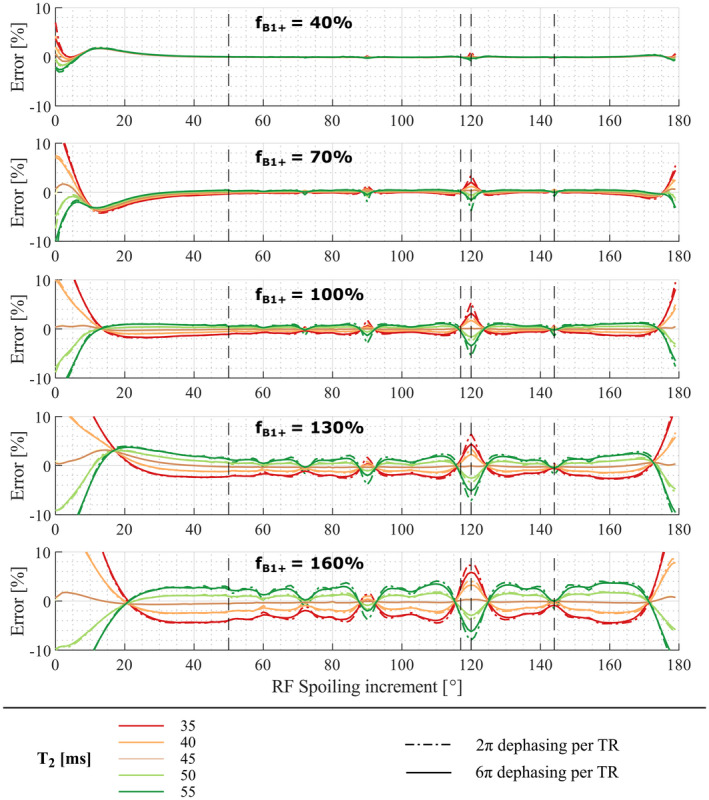
Residual errors after applying imperfect spoiling correction parameters derived with T_2_ = 45 ms and D = 0.8 µm^2^/ms to $T_{1}^{\text{app}}$. $T_{1}^{\text{app}}$ was estimated from data simulated with a true T_1_ time of 1.5 s, and a true T_2_ time ranging from 35 to 55 ms for *nPi* = 2π (dashed lines) or 6π (solid lines). Each row corresponds to a different $B_{1}^{+}$ efficiency, $f_{B_{1}^{+}}$, ranging from 40 to 160%. The error is minimized when the T_2_ used to estimate the correction parameters matches the true T_2_ (ie, 45 ms). Dashed vertical lines indicate the RF spoiling increments used in the in vivo acquisitions (ie, 50°, 117°, 120°,and 144°)

Correction factors derived from numerical simulations with T_2_ = 45 ms and D = 0.8 µm^2^/ms dramatically decreased the error in $T_{1}^{\text{app}}$ (c.f. Figures 1A and 2 middle row). However, the amplitude of the residual error was amplified by high $f_{B_{1}^{+}}$ and depended on whether the T_2_ used to derive the correction factors (45 ms) matched that of the simulation (Figure 2). For the commonly used *ϕ*_0_ of 50°, a discrepancy in T_2_ of 10 ms coupled with $f_{B_{1}^{+}}$ = 160% had a residual error of 2.7%.

Some RF spoiling increments were more robust to the choice of T_2_ and had lower residual error that further decreased by increasing the spoiler gradient moment. For the optimal combination of *ϕ*_0_ = 144° and *nPi* = 6π the correction parameters reduced the error to less than 0.8% for the full range of parameters investigated (Figure 2).

The particularly low T_2_ sensitivity of 144° motivated its use in subsequent experiments. 120° was additionally investigated because of its comparatively high sensitivity to all parameters. 50° and 117° were selected because they are the most commonly encountered increments.

### 3.3 Comparison between simulation and phantom experiment

Reference T_2_, T_1_ and ADC values in the phantom were estimated to be 78 ± 2 ms, 952 ± 14 ms and 1.71 ± 0.04 μm^2^/ms, respectively.

**FIGURE 3:**
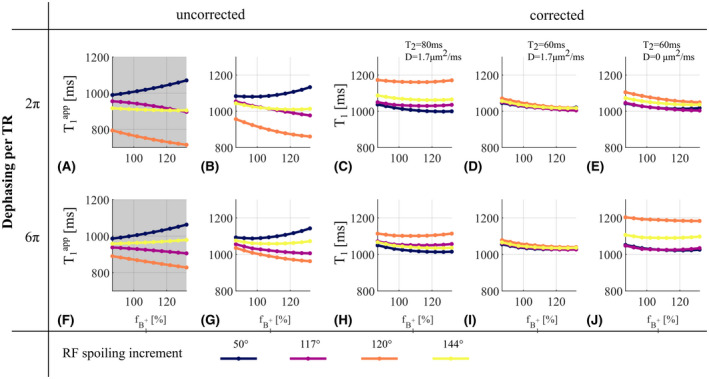
$T_{1}^{\text{app}}$ (A,B,F,G) and corrected T_1_ (C,D,E,H,I,J) in the phantom with $\phi_{0}\in {\left[50,117,120,144\right]}^{\circ }$, as a function of the $B_{1}^{+}$ efficiency, $f_{B_{1}^{+}}$. Dephasing across a voxel of 2π (A‐E) and 6π (F‐J) per TR are shown. A,F, Numerical simulations. Fixed parameters are: T_1_ = 950 ms, T_2_ = 80 ms, D = 1.7 µm^2^/ms. B‐E,G‐J, Acquisitions. Corrected T_1_ with correction factors from set 1 (C,H), set 2 (D,I), and set 3 (E,J) from Table 1A

The $f_{B_{1}^{+}}$ dependence of $T_{1}^{\text{app}}$ (Figure 3A,B,F,G) matched the numerical simulations. $T_{1}^{\text{app}}$ increased with $f_{B_{1}^{+}}$ for 50° (+ 50 ms) but decreased for 120° (−96 ms), and 117° (−77 ms), while 144° showed least sensitivity (−30 ms). The $f_{B_{1}^{+}}$ dependence of the increments of 120° and 144° was impacted by the increase of the spoiler gradient moment, whereas it had no observable impact for 117° and 50°.

$T_{1}^{\text{app}}$ maps were corrected with three sets of correction factors. The maps corrected with a T_2_ of 80 ms showed almost no $f_{B_{1}^{+}}$ dependence but had a *ϕ*_0_‐dependent offset (Figure 3C,H)_,_ which was removed when T_2_ was reduced to 60 ms (Figure 3D,I). However, when the correction factors were computed without accounting for diffusion, the corrected T_1_ again showed *ϕ*_0_‐dependent offsets_,_ particularly with a large spoiler gradient moment (Figure 3E,J).

### 3.4 Comparison between simulation and in vivo experiment

Reference T_2_, T_1_ and ADC value in the WM region used for subsequent analyses were estimated to be 47 ± 6 ms, 1260 ± 50 ms and 0.7 ± 0.1 µm^2^/ms, respectively.

**FIGURE 4:**
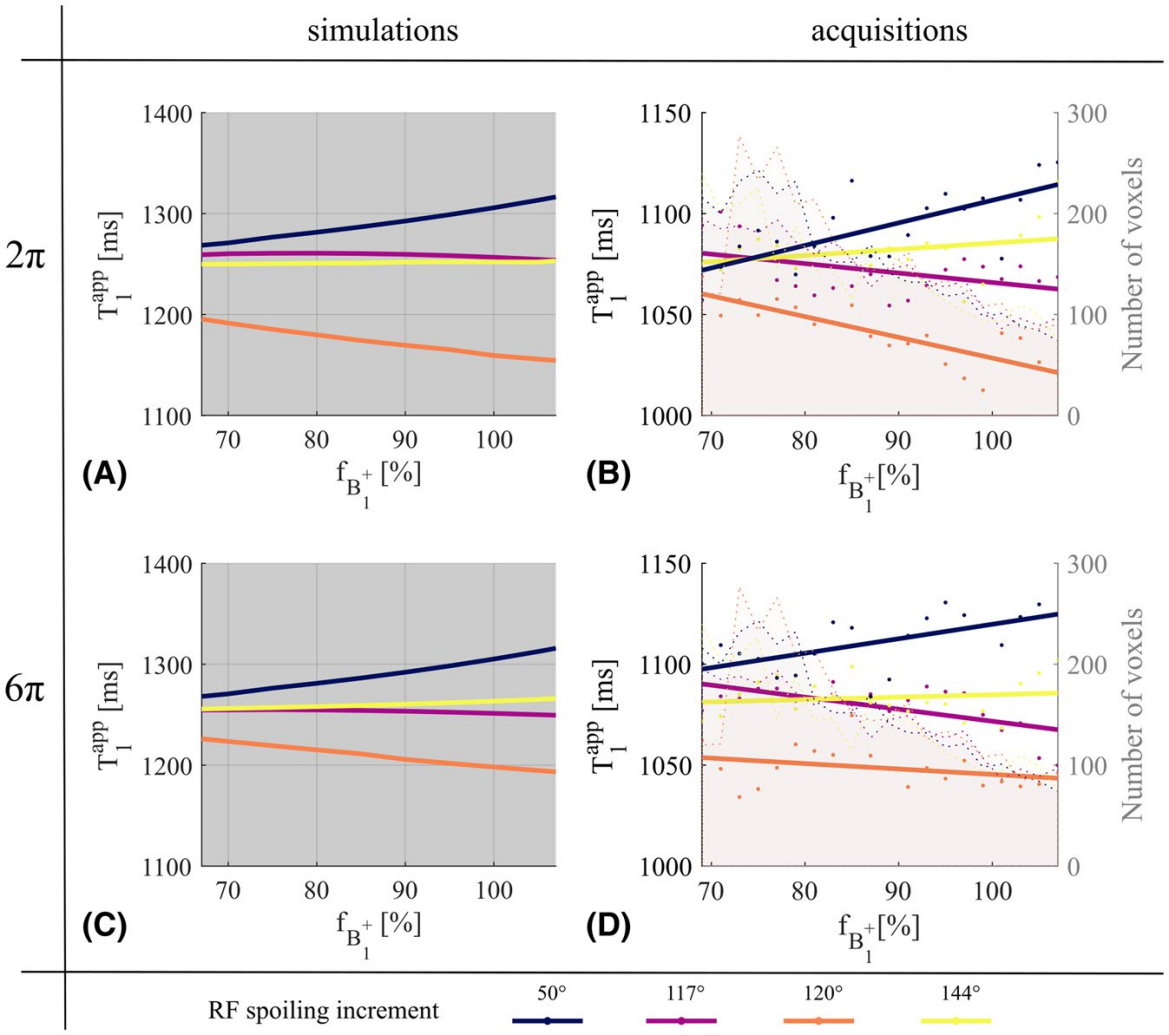
*In vivo*$T_{1}^{\text{app}}$, obtained with $\phi_{0}\in {\left[50,117,120,144\right]}^{\circ }$, as a function of the $B_{1}^{+}$ efficiency, $f_{B_{1}^{+}}$. Dephasing across a voxel of 2π (A,B) and 6π (C,D) per TR are shown. A,C, Numerical simulations. Fixed parameters are: T_1_ = 1250 ms, T_2_ = 45 ms, D = 0.8 µm^2^/ms. B,D, Acquisitions, and linear fitting for illustration purposes. The number of voxels included in each bin is depicted by the shaded background of each graph for each *ϕ_0_*

The dependence of $T_{1}^{\text{app}}$ on $f_{B_{1}^{+}}$ (Figure 4A,C) predicted by simulation was observed *in vivo* (Figure 4B,D). *ϕ*_0_ of 50° and 120° were most sensitive to $f_{B_{1}^{+}}$ with $T_{1}^{\text{app}}$ varying by +41 ms or −40 ms, respectively, between 65 and 110% efficiency. The variation was 24 ms for 117° and 5 ms for 144°. As predicted, increasing the gradient spoiler moment had the biggest impact on 120°, for which the $T_{1}^{\text{app}}$ variation decreased to −15 ms over the range of $f_{B_{1}^{+}}$.

**FIGURE 5:**
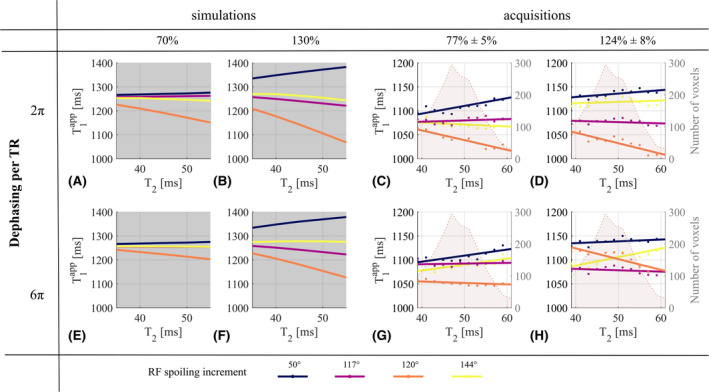
*In vivo*$T_{1}^{\text{app}}$, obtained with $\phi_{0}\in {\left[50,117,120,144\right]}^{\circ }$, as a function of T_2_. Dephasing across a voxel of 2π (A‐D) and 6π (E‐H) per TR are shown. Original (×1) (A,C,E,G) and high (×1.6) (B,D,F,H) transmitter reference voltage are shown. A,B,E,F, Numerical simulations. Fixed parameters are: T_1_ = 1250 ms, D = 0.8 µm^2^/ms, $f_{B_{1}^{+}}=70\text{\%}\;$, and 130% as measured in the transmit field map in the slice of interest. C,D,G,H, Acquisitions, and linear fitting for illustration purposes. The number of voxels included in each bin is depicted by the shaded background of each graph for each *ϕ_0_*

Simulations predicted that, as T_2_ increased, $T_{1}^{\text{app}}$ would be under‐estimated for *ϕ*_0_ = 120° or slightly over‐estimated for *ϕ*_0_ = 50° (Figure 5). A small T_2_‐dependence was predicted for 117° and even less so for 144°. These dependencies were predicted to be accentuated when $f_{B_{1}^{+}}$ increased to 130% and to be reduced when *nPi* was increased from 2π to 6π. Most of those predictions were observed *in vivo*: over the T_2_ range, with 2π gradient spoiling, $T_{1}^{\text{app}}$ decreased by 51 ms for 120° and increased by 30 ms for 50°. This reduced to 12 and 25 ms, respectively, for 6π. The variation of $T_{1}^{\text{app}}$ for 117° and 144° did not exceed 20 ms. Discrepancies between simulations and acquisitions occurred in the case of high $f_{B_{1}^{+}}$ and gradient spoiling (Figure 5H). $T_{1}^{\text{app}}$ showed the predicted reduction with increasing T_2_ for 120°, but an unpredicted offset. An unpredicted increase was also observed for 144° while the increased T_2_‐dependence predicted for 50° was not observed (Figure 5D,H).

### 3.5 Impact of correction parameters in vivo

**FIGURE 6:**
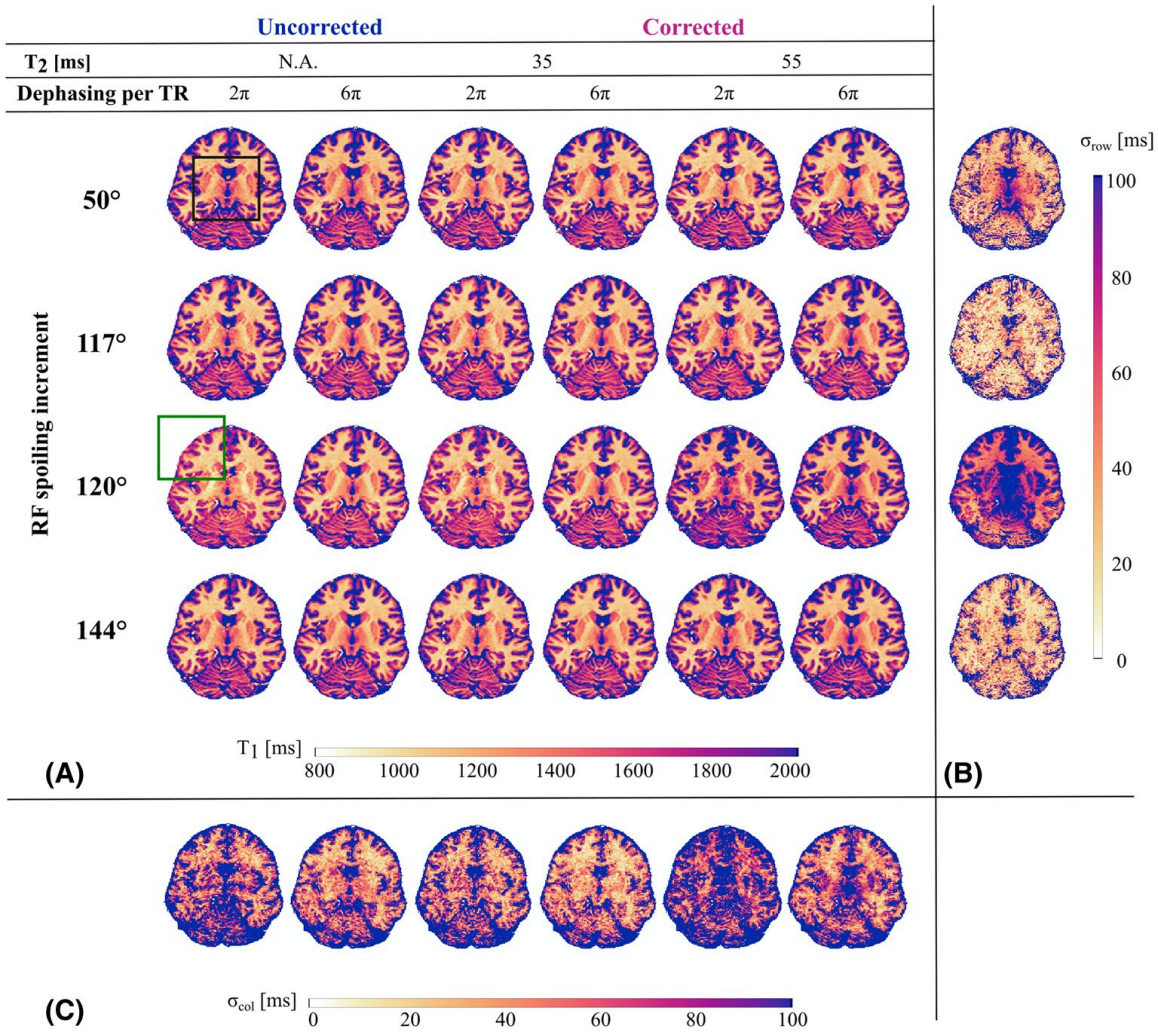
A, Axial view from T_1_ maps obtained with nominal $f_{B_{1}^{+}}$ (ie, no reference voltage manipulation) and *nPi* = 2π (columns 1, 3, and 5) or 6π (columns 2, 4, and 6) for each RF spoiling increment (rows). These are presented before (ie, $T_{1}^{\text{app}}$, columns 1 and 2) and after correction for imperfect spoiling using a fixed D of 0.8 µm^2^/ms and a T_2_ of either 35 ms (columns 3 and 4) or 55 ms (columns 5 and 6). Black and green boxes highlight areas particularly affected by changing *nPi* or applying correction factors. B, Maps of *σ*_row_, the voxel‐wise SD of T_1_ across conditions for a given RF spoiling increment (ie, along rows in (A)). C, Maps of σ_col_, the voxel‐wise SD of T_1_ across RF spoiling increments for a given condition (ie, along columns in (A))

Although the T_1_ maps with different spoiling conditions, with and without correction for imperfect spoiling, were qualitatively similar (Figure 6), some distinct contrast differences were observed, for example, between GM and cerebrospinal fluid (green box) and within the basal ganglia (black box). The within‐increment SD (*σ_row_*) across T_1_ maps revealed particularly high sensitivity to spoiling condition and the T_2_ used to derive the correction parameters (c.f. Figure 6B *ϕ*_0_ of 50° or 120° versus 117° or 144°). The T_1_ maps converged (low *σ_col_*, Figure 6C) when sufficient gradient spoiling (*nPi* = 6π) was combined with a T_2_ of 35 ms to derive the correction parameters, but diverged (high *σ_col_*, Figure 6C) when low spoiling (*nPi* = 2π) was combined with no correction for imperfect spoiling or one based on a T_2_ of 55 ms.

**FIGURE 7:**
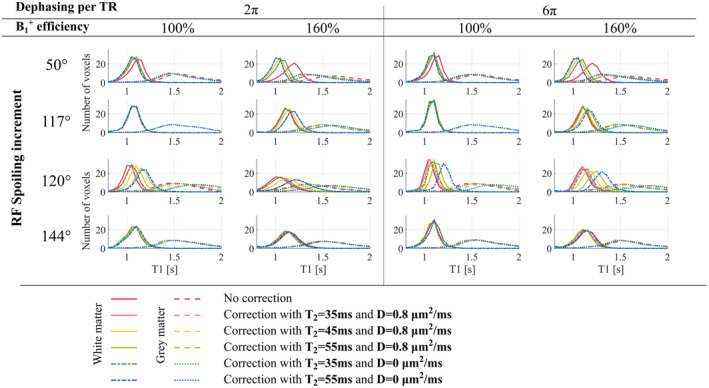
Histograms of the estimated T_1_ times for WM and GM without (ie, $T_{1}^{\text{app}}$) and with correction for imperfect spoiling. Five sets of correction factors were computed based on different T_2_ times and diffusion coefficients and applied separately. Only those voxels with $f_{B_{1}^{+}}$ between 95% and 105% (measured with no reference voltage manipulation) were included in the analysis

The impact of correcting for imperfect spoiling increased with $f_{B_{1}^{+}}$ (Figure 7). The correction induced the largest shifts in T_1_ for *ϕ*_0_ of 50° (Figure 7), especially when $f_{B_{1}^{+}}$ = 160%. The higher variance observed across conditions for this increment (Figure 6B) also exhibits a pattern consistent with the $f_{B_{1}^{+}}$ profile. *ϕ*_0_ = 117° only benefited from correction at high $f_{B_{1}^{+}}$.

Corrected T_1_ times were highly dependent on the T_2_ used for the correction, especially when $f_{B_{1}^{+}}$ was 160%. A global T_2_ time of 35 ms minimized dependence on *ϕ*_0_ (Figure 6C). *ϕ*_0_ = 120° was additionally dependent on D regardless of experimental conditions. *ϕ*_0_ = 144° was exceptional in that the corrected T_1_ times were effectively independent of the tissue properties (T_2_ and D) used to determine the correction parameters.

### 3.6 Artifact dependence on spoiling conditions

**FIGURE 8:**
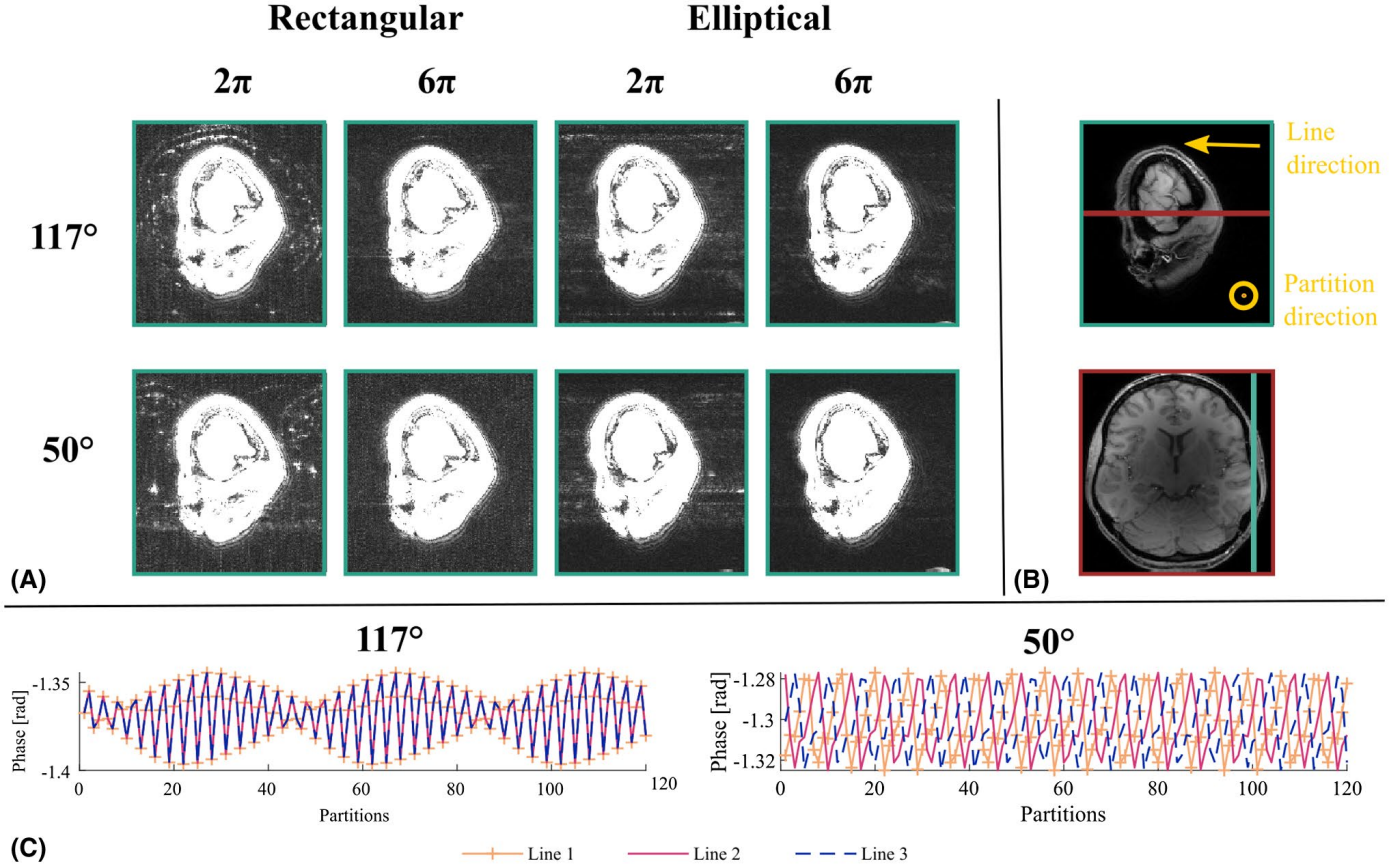
A, Sagittal view of T_1_‐weighted images acquired with rectangular or elliptical k‐space sampling using different spoiling conditions. The images have been windowed to highlight background signal. B, The same sagittal slice and an axial slice are shown windowed to visualize the brain. The turquoise line in the axial view indicates the sagittal position while the red line on the sagittal view indicates the axial position. The two phase‐encoding directions (lines and partitions) are indicated in the sagittal view. C, Numerical simulations of the impact of partial voluming on the phase of the signal across lines and partitions (rectangular sampling case, all partitions are acquired before incrementing the line) for each RF spoiling increment

The weighted images used to calculate T_1_ were affected by background artifacts when *nPi* = 2π (Figure 8A). With rectangular k‐space sampling, the artifact manifested as a coherent alias of brain edges. Its location depended on *ϕ*_0_: the replica was shifted in the partition direction for 117°, but in both the partition and line directions for 50°. While still visible, the artifact was more diffuse with elliptical sampling (Figure 8A). Numerical simulations with rectangular k‐space sampling based on a mixed population of isochromats (different resonance frequencies) showed phase variation across TR (Figure 8C). *ϕ*_0_ = 117° produced a periodic phase variation across partitions consistent with the observed position of the ghost artifact in the field‐of‐view along this direction. *ϕ*_0_ = 50° resulted in phase variation in both the lines and partitions directions, again consistent with the empirical data.

## 4 DISCUSSION

We have demonstrated a complex relationship between the estimated T_1_ and the spoiling regime within the context of the 3D VFA approach. Our simulations (Figure 1) indicate that no RF spoiling increment would lead to $T_{1}^{\text{app}}$ matching the true T_1_ across all conditions (ie, sequence choices, T_2_ times, and diffusion coefficients). Furthermore, the simulations show that even the fullest effects of diffusion achieved in a multi‐echo acquisition are insufficient to achieve perfect spoiling of the transverse magnetisation.

The sensitivity to imperfect spoiling effects will depend on the specifics of the protocol used. It has recently been suggested that the maximum flip angle should be minimized to mitigate spoiling‐induced errors.^37^ However, for the TR and target T_1_ range considered here, this would compromise the precision of the T_1_ estimates by as much as 50%.^38^ An alternative would be to reduce both flip angles and TR, which could be achieved by adopting a single echo protocol. However, simulations suggest that the sensitivity of such an approach would be on a par with that observed in the protocol used here (c.f. Figure 1 and Supporting Information Figure S2). Combining a single echo approach with a long TR and using the time to impart extensive gradient spoiling is predicted to reduce the sensitivity of T_1_ estimates to imperfect spoiling, even in the case of high flip angles (Supporting Information Figure S2). However, unlike the multi‐echo MPM protocol adopted here, single echo protocols prevent $T_{2}^{*}$ from being concurrently estimated, and prevent extrapolation to TE = 0 ms, which aims to remove bias introduced by flip angle‐dependent $T_{2}^{*}$ decay^35^, ^39^ from the T_1_ estimates. Slice‐selective 2D acquisitions would naturally achieve a long TR and improved spoiling behaviour, but suffer limitations such as MT effects and imperfect slice profiles that lead to flip angle‐dependent^40^ bias in the T_1_ estimates.

In the context of the MPM protocol investigated here $T_{1}^{\text{app}}$ was most sensitive to $f_{B_{1}^{+}}$, which could lead to over‐estimation of the true T_1_ by as much as 30%. Despite accounting for $f_{B_{1}^{+}}$ inhomogeneity in both simulations and acquisitions (Equation 1), $T_{1}^{\text{app}}$ continued to depend on $f_{B_{1}^{+}}$ in an increment‐specific manner. Some *ϕ*_0_, such as the commonly used 50°, were particularly sensitive (Figures 1,3 and 4), although the error can be markedly reduced by post‐hoc correction for imperfect spoiling (Figures 2,3 and 6). Since the correction factors are a function of both $T_{1}^{\text{app}}$ and $f_{B_{1}^{+}}$ (Equation 3), they rely on accurate estimation of the true $f_{B_{1}^{+}}$. Any error will propagate through to the corrected T_1_ time. It may then be appealing to select a *ϕ*_0_ that exhibits lower sensitivity to $f_{B_{1}^{+}}$, for example, 117° or 144° (Figures 1 and 3). However, the sensitivity of $T_{1}^{\text{app}}$ to inaccurate or imprecise definition of the flip angles ($f_{B_{1}^{+}}$ in Equation 1) would remain.^41^

**FIGURE 9:**
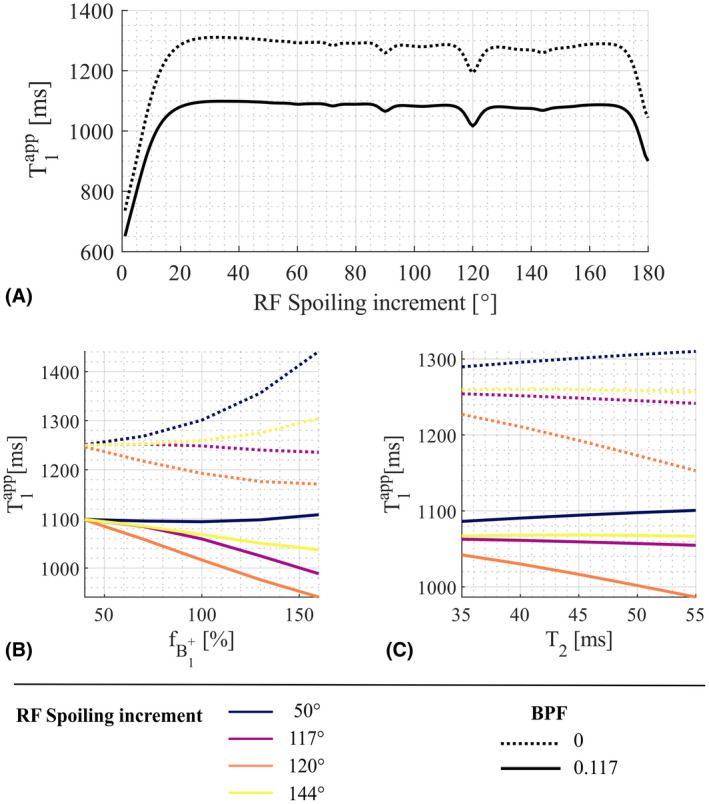
Numerical simulations without (BPF = 0, dashed line) and with (BPF = 0.117, solid line) a bound pool fraction leading to MT. The exchange rate from the free to the bound pool was 4.3 s^−1^,^44^ and thermal equilibrium was assumed. The BPF was 0.117^44^ and the diffusion coefficient was 0.8 µm^2^/ms. A, $T_{1}^{\text{app}}$ for *ϕ*_0_ from 1 to 180°, T_2_ = 45 ms, T_1_ = 1.25 s (for both pools), and $f_{B_{1}^{+}}$ of 100%. The total dephasing per TR was set to 6π. B‐C, $T_{1}^{\text{app}}$ as a function of $f_{B_{1}^{+}}$ and T_2_ time, respectively, for *ϕ*_0_ = 50°, 117°, 120°, and 144°

Other sources of $f_{B_{1}^{+}}$ dependence may affect the accuracy of the T_1_ estimate, and were reduced as much as possible in this work:

1. MT effects, proportional to the square of the amplitude of the transmit field, have been shown to bias $T_{1}^{\text{app}}$ ^37^, ^42^, ^43^ and can be expected to be a more significant problem at 7T. Experimentally, the MT effect was limited by the use of rectangular pulses with matched power for the PD‐ and T_1_‐weighted acquisitions. Nonetheless, residual dependence may remain and may underlie the offset between the T_1_ measured in WM with the IR‐SE‐EPI (1260 ms) and VFA (~1100 ms) approaches. MT effects were not included in any of our simulations of the steady‐state signal since it depends on a number of unknown, spatially varying tissue properties. However, we can estimate the impact of MT effects in the present context for WM using the EPG‐X framework (https://github.com/mriphysics/EPG‐X).^44^, ^45^ Assuming a bound pool fraction (BPF) of 0.117,^44^ thermal equilibrium with an exchange rate from free to bound pool of 4.3 s^−1^,^44^ and a T_1_ time of 1.25 s for each pool, the MT effect caused $T_{1}^{\text{app}}$ to decrease as $f_{B_{1}^{+}}$ increased. Depending on the RF spoiling increment used, this bias could either counteract (eg, *ϕ*_0_ = 50°) or accentuate (eg, *ϕ*_0_ = 120°) the $f_{B_{1}^{+}}$ sensitivity induced by imperfect spoiling (Figure 9B). However, the MT effect was largely independent of the RF spoiling increment (Figure 9A). A method has recently been proposed to counteract MT effects at the time of acquisition.^43^
2. $T_{2}^{*}$ has previously been shown to depend on the excitation flip angle.^35^ If differential $T_{2}^{*}$ weighting exists in the SPGR signals Equation (1) will lead to biased T_1_ estimates. Unlike single echo approaches, the multi‐echo approach adopted here allows extrapolation to TE = 0 ms to limit this source of $f_{B_{1}^{+}}$‐dependent bias.
3. The approximations implemented by default in the hMRI toolbox offer flexibility but introduce $f_{B_{1}^{+}}$‐dependent bias when the assumptions are violated at higher flip angle.^13^ Matched TR across the SPGR acquisitions permitted $T_{1}^{\text{app}}$ to be estimated without approximation^14^ circumventing this potential source of bias.

At 7T, T_2_ shortens increasing the feasibility of fully spoiling the transverse magnetisation. Any T_2_‐dependence is an important consideration since it can lead to spatially varying, microstructurally driven error in T_1_ estimation. Both simulation and experiment showed that some RF spoiling increments are particularly sensitive to T_2_ (Figure 5) and that the error increases in regions of high $f_{B_{1}^{+}}$. Analysis of the EPGs can provide some insights into these observations (Supporting Information S4, Supporting Information Figures S5 and S6). However, incorporating knowledge of T_2_ to correct this error is difficult since accurate quantification is challenging and time‐consuming, particularly at high resolution, and can also depend on factors like $f_{B_{1}^{+}}$ and slice profiles.^46^, ^47^ Here, we have observed the T_2_‐dependence of $T_{1}^{\text{app}}$ by measuring T_2_ with a single‐echo spin‐echo EPI technique to maximise accuracy and minimize confounding factors. The choice of T_2_ used to derive the correction factors is particularly important for the commonly used *ϕ*_0_ = 50°, which exhibits higher T_2_ sensitivity (Figures 3, 6, and 7). Although the post‐hoc correction for imperfect spoiling is not voxel‐specific, global T_2_ times of 35 ms *in vivo* and 60 ms in phantom, resulted in the convergence of the corrected T_1_ distributions regardless of their acquisition conditions (Figures 3, 6, and 7). However, these T_2_ times were smaller than literature values for *in vivo* WM at 7T^28^ and those estimated with the reference protocol (T_2_ of 48 ± 6 ms and 78 ± 2 ms *in vivo* and in phantom, respectively). Note that using an array of T_2_ times to match those expected *in vivo* will have the same compromise effect whereby error may increase or decrease depending on the true T_2_.

The issue of T_2_‐dependence in the estimated T_1_, including after correction, has been highlighted previously at 3T.^12^, ^19^ The manifestation of imperfect spoiling at 3T differs to 7T most notably by a greater dependence on the gradient spoiling and diffusional effects at 3T (see Supporting Information Figures S3 and S4 for a 3T analysis). Baudrexel et al^19^ proposed an alternative correction method to the one used here, which adjusts the flip angle to account for imperfect spoiling before estimating T_1_. The T_2_‐dependence of that technique was not compared to the method used here, but in phantoms with variable T_2_, showed a residual deviation of 1 to 3%. As an alternative to estimating T_1_ using an analytical solution of the Ernst equation, Heule et al^12^ proposed a numerical minimization approach that does not rely on perfect spoiling. The approach performed well in comparison to the correction technique adopted here, particularly in terms of reducing theoretical sensitivity to T_2_. However, the residual error increased with the diffusion coefficient and the moment of the spoiler gradient. Here, we have shown that a large spoiler moment is beneficial not only to reduce $T_{1}^{\text{app}}$ sensitivities, but also to minimize artifacts due to partial voluming (Figure 8). The ghost artifacts we observed, and explained by numerical simulation, are another consequence of imperfect spoiling previously highlighted by Nielsen and Noll^31^ and an important consideration when designing a protocol.

At 7T, simulations incorporating the diffusion effect, due to both the multi‐echo readout and the spoiler gradient, demonstrated a detectable effect on $T_{1}^{\text{app}}$ when using *in vivo* T_2_ times and clinically feasible spoiler moments. In phantom, where the ADC was estimated to be 1.71 µm^2^/ms, the sensitivity to $f_{B_{1}^{+}}$ depended on the spoiler gradient moment, and it was necessary to include the diffusion effect when determining the correction factors to obtain consistent $T_{1}^{\text{app}}$ across φ_0_, especially for the large spoiler gradient condition. In healthy *in vivo* tissue, the estimated ADC was lower (0.7 µm^2^/ms) in line with literature.^29^, ^30^ The sensitivity to this parameter over the range of likely values was generally small (<1%), even for *nPi* = 6π, which required a net moment of 70.5 mT/m.ms at 1 mm resolution. Nonetheless, the effect of diffusion was observed *in vivo* and the T_2_‐dependence was reduced for *ϕ*_0_ of 50° and 120°, when *nPi* was increased to 6π, in line with the simulations. Including the diffusion effect in the correction factors only had an appreciable impact with φ_0_ = 120°. However, the higher the spoiler gradient moment, the higher the expected sensitivity to the diffusion coefficient (Figures 1I, Supporting Information Figures S2 and S3I).

RF spoiling increments of 50° and 117° are commonly used. 50° has the advantage of being located in a stable region^17^ but, as shown here, may not be the most suitable because of its high sensitivity to $f_{B_{1}^{+}}$ and T_2_. 117° is known for being close to perfect spoiling conditions. However, it is shown here that despite reducing $T_{1}^{\text{app}}$ error, residual T_2_‐dependence remains, especially at high $f_{B_{1}^{+}}$. *ϕ*_0_ = 144° shows appealing robustness to T_2_ with clinically feasible spoiler moments, making it a good candidate for VFA‐based T_1_ measurements. However, a broader histogram (Figure 7) and larger than predicted T_2_‐dependence (Figure 5E‐H) were measured *in vivo* for this increment, meaning that we cannot exclude the possibility that it may be a less stable choice.^17^

### 4.1 Limitations

Although efforts were made to remove confounding effects, such as anatomical variability, some discrepancies were observed between simulations and experiments (Figure 5). In addition to the issue of MT effects discussed earlier, the observed discrepancies may come from residual variance in T_1_ or diffusion properties, or other microstructural features not well modeled by single pool simulations, for example, myelin water.

Some further limitations warrant discussion. This work has been performed at 7T using only a small number of protocols with many fixed parameters, for example, resolution, TR and number of echoes. Nonetheless, good agreement was observed between simulations and acquisitions indicating that the same framework could be used to investigate other protocols.

The resolution used is relatively low for 7T imaging (1 mm isotropic) but was required to maintain a tolerable scan time per session given the number of factors probed ($f_{B_{1}^{+}}$, *nPi*, *ϕ*_0_). With high resolution, the diffusion effect can be expected to increase due to the larger moment of the readout gradients, while the risk of partial voluming will concurrently reduce.

The sensitivity of $T_{1}^{\text{app}}$ to each of the sequence parameters and tissue properties was investigated for a limited range of values. However, these were selected to encompass the range expected in the context of neuroimaging at 7T. The sensitivity may increase in pathology, but again the validated framework presented here could be used to determine optimal protocol strategies.

## 5 CONCLUSIONS

We conclude by returning to the questions posed in the introduction:

1. How do RF and gradient spoiling interact and which combination maximizes the accuracy and precision of T_1_ mapping with the VFA approach?

The interaction between RF and gradient spoiling is complex. The error in $T_{1}^{\text{app}}$ depends not only on *ϕ*_0_, but also on $f_{B_{1}^{+}}$ and T_2_ times, as does its sensitivity. The impact that gradient spoiling has on the error and sensitivity also depends on *ϕ*_0_. Increasing the net gradient‐induced dephasing per TR reduces the dependence on *ϕ*_0_, except in terms of sensitivity to the diffusion coefficient (Figure 1). Since no combination achieves perfect spoiling, the preferred approach may be to maximise the robustness of $T_{1}^{\text{app}}$ to other parameters that are fixed in post‐hoc correction, principally T_2_. Larger spoiler gradient moment has the additional benefit of improving image quality.

- What impact does the diffusion effect have when considering clinically feasible gradient moments and the impact of the full readout?

Including the full diffusion effect of a multi‐echo readout has a comparatively small impact at 7T relative to 3T for healthy tissues (Supporting Information Figures S3 and S4). Correction factors accounting for diffusion are especially important at lower field strengths due to longer T_2_ or in pathology for higher diffusion coefficient, and can now be estimated via the hMRI toolbox (hmri.info^20^).

- What are the limitations of applying simulation‐derived correction factors to recover the true T_1_ from $T_{1}^{\text{app}}$?

The main limitation of post‐hoc correction is the need to specify a single T_2_ time, leading to residual T_2_‐dependence. Combining *ϕ*_0_ = 144° with moderate gradient spoiling minimizes the sensitivity of $T_{1}^{\text{app}}$ to T_2_.

## Supporting information

**FIGURE S1** (A) Positioning of the slices used to obtain reference T_1_ (B,E) and T_2_ (C,F) times using single slice, single echo, spin echo EPI acquisitions. Reference ADC estimates (D,G) from the same slices are also shown. The more superior slice (yellow) had lower $f_{B_{1}^{+}}$ variance and was therefore used to investigate the T_2_ dependence. The more inferior slice (red) had greater $f_{B_{1}^{+}}$ variance and was therefore used to investigate the $f_{B_{1}^{+}}$ dependence
**FIGURE S2** Sensitivity of $T_{1}^{\text{app}}$ to $B_{1}^{+}$ efficiency (A), the true T_2_ time (B,C), the true T_1_ time (D,E) and the diffusion coefficient (F‐G) of three single‐echo protocols: Protocol 1 (blue), 2 (red) and 3 (yellow). The sensitivity to T_1_, T_2_ and D are computed in two conditions: $B_{1}^{+}$ efficiency of 100% or 160%
**FIGURE S3** Numerical simulations, for each spoiling condition, of $T_{1}^{\text{app}}$ error in two specific cases: (A) T_1_ = 1250 ms, D = 0.8 µm^2^/ms, T_2_ = 65 ms and $f_{B_{1}^{+}}$ = 100%, (B) T_1_ = 0.75 s, D = 1.0 µm^2^/ms, T_2_ = 55 ms and $f_{B_{1}^{+}}$ = 130%. Sensitivity of $T_{1}^{\text{app}}$ to $f_{B_{1}^{+}}$ (C), the true T_2_ time (D‐E), the true T_1_ time (F‐G) and the true diffusion coefficient (H‐I). The sensitivity to T_1_, T_2_ and D are computed in two conditions: $B_{1}^{+}$ efficiency of 100 % (D‐F‐H) or 130 % (E‐G‐I)
**FIGURE S4** Acquisitions and numerical simulations at 3T for RF spoiling increments of 30°, 72°, 117°, 120° and 137°. T_1_ before (ie, $T_{1}^{\text{app}}$) and after correction for imperfect spoiling with correction factors determined assuming T_2_ = 65 ms and D = 0.8 µm^2^/ms or ignoring diffusion. Simulations (left): true T_1_ times are indicated by a solid black line at 1 s and 1.5 s. In vivo acquisitions (right): distribution of T_1_ times in GM and WM where $B_{1}^{+}$ efficiency was between 90% and 110%
**FIGURE S5** EPG diagrams, before (A, C) and after (B, D) incorporating diffusion. The diagrams depict the population amplitude of transverse, $\left|F_{n}\right|$ and longitudinal, $\left|Z_{n}\right|$, configuration states with n the degree of dephasing ϕ=2π. The simulations used a T_1_ of 1000 ms, T_2_ of 80 ms and a diffusion coefficient of 1.7 μm^2^/s with a flip angle of 6° and a TR of 19.5 ms. Note that for visualisation purposes only a subset of the EPG diagrams are shown: 0 < *n* ≤ 20 for the dephasing transverse configuration states, 0 > *n* ≥ −20 for the rephasing transverse magnetization and 0 ≤ *n* ≤ 40 for the longitudinal states. Higher order longitudinal and rephasing states are more populated for the RF spoiling increments of 120° and 144°. Since higher order states are especially attenuated by diffusion, incorporating this effect has the most appreciable impact on these increments. It can also be seen that sufficient pulses are incorporated to reach a steady state, which is arrived at comparatively quickly for all increments
**FIGURE S6** Intra‐voxel magnetisation distribution derived from the steady‐state EPG coefficients for each increment (A), and the population amplitudes of the first six rephasing transverse configuration states (B). Four cases are shown: two nominal flip angles (6° and 26° corresponding to the PDw and T1w acquisitions of this study) and two T_2_ times (60 ms and 80 ms). All other simulation settings are as in Supporting Information Figure S5. The net SPGR echo‐forming signal (ie, $F_{0}$ state) is projected onto the transverse plane in (A) with an artificial phase dispersion added to aid visualisation
