# Supplementary material for: Imperfect spoiling in variable flip angle T1 mapping at 7T: Quantifying and minimizing impact
Source: Magn Reson Med. 2021 Mar 1;86(2):693–708. doi: 10.1002/mrm.28720 (PMC8436769; doi:10.1002/mrm.28720)
Supplement: Supplementary file 1 — FIGURE S1 (A) Positioning of the slices used to obtain reference T1 (B,E) and T2 (C,F) times using single slice, single echo, spin echo EPI acquisitions. Reference ADC estimates (D,G) from the same slices are also shown. The more superior slice (yellow) had lower fB1+ variance and was therefore used to investigate the T2 dependence. The more inferior slice (red) had greater fB1+ variance and was therefore used to investigate the fB1+ dependence FIGURE S2 Sensitivity of T1app to B1+ efficiency (A), the true T2 time (B,C), the true T1 time (D,E) and the diffusion coefficient (F‐G) of three single‐echo protocols: Protocol 1 (blue), 2 (red) and 3 (yellow). The sensitivity to T1, T2 and D are computed in two conditions: B1+ efficiency of 100% or 160% FIGURE S3 Numerical simulations, for each spoiling condition, of T1app error in two specific cases: (A) T1 = 1250 ms, D = 0.8 µm2/ms, T2 = 65 ms and fB1+ = 100%, (B) T1 = 0.75 s, D = 1.0 µm2/ms, T2 = 55 ms and fB1+ = 130%. Sensitivity of T1app to fB1+ (C), the true T2 time (D‐E), the true T1 time (F‐G) and the true diffusion coefficient (H‐I). The sensitivity to T1, T2 and D are computed in two conditions: B1+ efficiency of 100 % (D‐F‐H) or 130 % (E‐G‐I) FIGURE S4 Acquisitions and numerical simulations at 3T for RF spoiling increments of 30°, 72°, 117°, 120° and 137°. T1 before (ie, T1app) and after correction for imperfect spoiling with correction factors determined assuming T2 = 65 ms and D = 0.8 µm2/ms or ignoring diffusion. Simulations (left): true T1 times are indicated by a solid black line at 1 s and 1.5 s. In vivo acquisitions (right): distribution of T1 times in GM and WM where B1+ efficiency was between 90% and 110% FIGURE S5 EPG diagrams, before (A, C) and after (B, D) incorporating diffusion. The diagrams depict the population amplitude of transverse, Fn and longitudinal, Zn, configuration states with n the degree of dephasing ϕ=2π. The simulations used a T1 of 1000 ms, T2 of 80 ms and a diffusion coefficient of [file MRM-86-693-s001.pdf]

Supporting Information 1

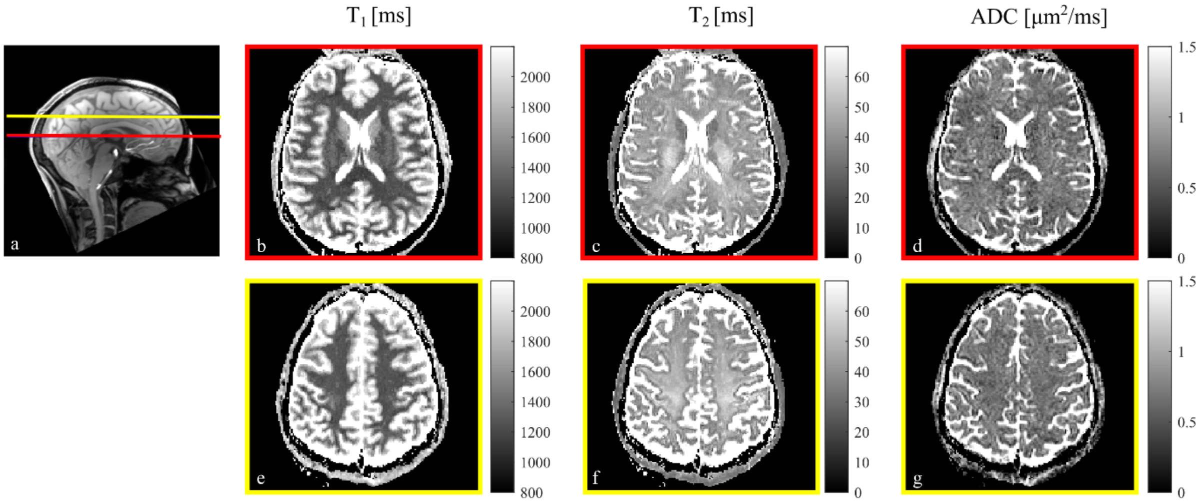

Supporting information Figure S1: (a) Positioning of the slices used to obtain reference T_1 (b,e) and T_2 (c,f) times using single slice, single echo, spin echo EPI acquisitions. Reference ADC estimates (d,g) from the same slices are also shown. The more superior slice (yellow) had lower $f_{B_1^+}$ variance and was therefore used to investigate the T_2 dependence. The more inferior slice (red) had greater $f_{B_1^+}$ variance and was therefore used to investigate the $f_{B_1^+}$ dependence.

Supporting information 2: Single echo protocols

Two single-echo protocols with large spoiler gradients were simulated to evaluate the effect of imperfect spoiling in maximally spoiled conditions, i.e. where the majority of the TR is spent spoiling the magnetisation as opposed to reading it out in the multi-echo fashion presented in the main manuscript. Those protocols are also compared to a single-echo protocol with a short TR, hence lower gradient spoiling moment. Key parameters were:

- **Protocol 1:** TR=19.5ms; flip angles 4°/15° ; total dephasing per TR of 50π
- **Protocol 2:** TR=19.5ms; flip angles 6°/26° ; total dephasing per TR of 48π
- **Protocol 3:** TR=10.5ms; flip angles 4°/15° ; total dephasing per TR of 6π

Note that the longer pulse duration of the 26 degree flip angle led to reduced time for gradient spoiling and hence the reduction from 50 to 48π. Simulation results are presented in Supporting Information Fig.S2.

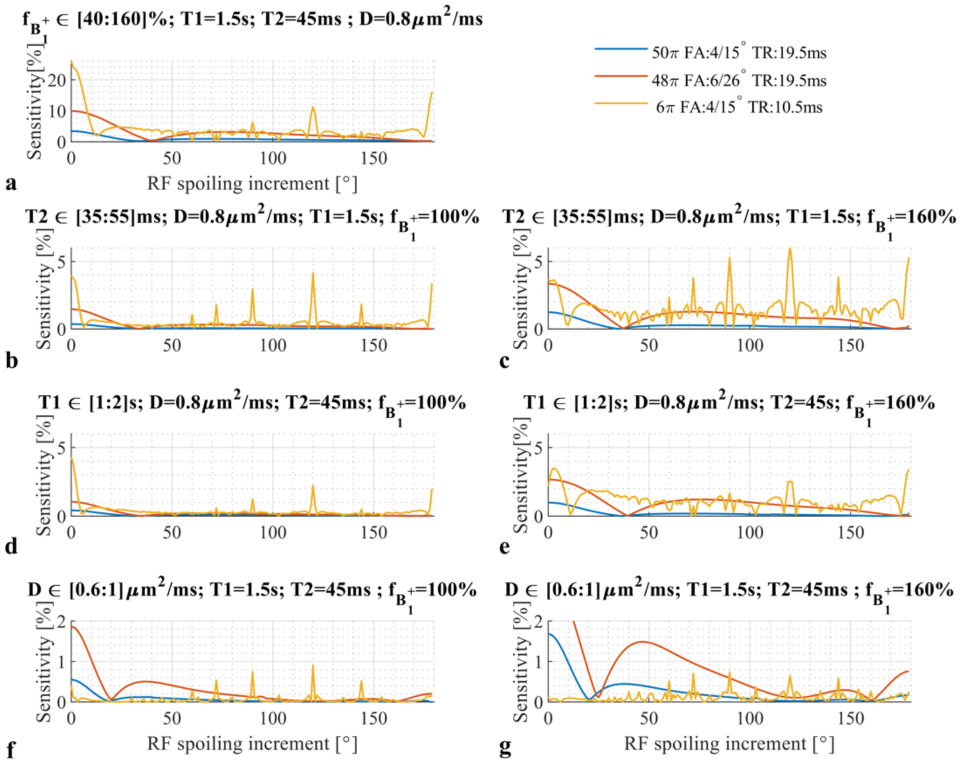

Supporting information Figure S2: Sensitivity of T_1^{app} to B_1^+ efficiency (a), the true T_2 time (b,c), the true T_1 time (d,e) and the diffusion coefficient (f-g) of three single-echo protocols: Protocol 1 (blue), 2 (red) and 3 (yellow). The sensitivity to T_1, T_2 and D are computed in two conditions: B_1^+ efficiency of 100% or 160%.

With equivalent TR, the sensitivity to each parameter, except the diffusion coefficient, was reduced for both single-echo protocols relative to the multi-echo protocols due to the extensive gradient spoiling, and the use of lower flip angles in the case of **Protocol 1**. Although **Protocol 1** shows close to perfect spoiling behaviour, and therefore least sensitivity to sequence settings and tissue properties, this is at the cost of a 50% increase in the expected variance of the T_1 estimates due to greater noise propagation as a result of the sub-optimal choice of flip angles *(1)*. While reducing the TR of this protocol will decrease the expected variance of the T_1 estimate, it also degrades the spoiling conditions due to the lower gradient moment per TR (**Protocol 3**). Furthermore, the single-echo nature of these protocols prohibits the estimation of T_2* and the extrapolation to TE=0ms.

Supporting information 3: Imperfect spoiling in variable flip angle T_1 mapping at 3T

<u>Numerical simulations</u>

Additional simulations were performed using 3T-relevant parameters. The SPGR signal intensity was simulated for flip angles of 4° and 25° and a TR of 18ms. The diffusion-driven spoiling effect imparted by the readout and spoiler gradients, applied along the same axis and leading to a total dephasing per TR of nPi, varying from 2π to 6π with an increment of 2π across a voxel, was incorporated. The simulations spanned the same range of values for the diffusion coefficient, B_1^+ efficiency and RF spoiling increments as used at 7T: D varying from 0.6μm²/ms to 1μm²/ms with an increment of 0.1μm²/ms, ϕ_0 varying from 0° to 179° with a step of 1° and $f_{B_1^+}$ varying from 40% to 160% with a step of 30%. However, the range of T_1 and T_2 times were adjusted to be 3T-specific: T_1 between 750ms and 1750ms with a step of 250ms and T_2 between 55ms and 75ms with a step of 5ms. The same evaluation metrics used at 7T were used to assess the bias in T_1^{app} and its sensitivity to each of the parameters. Results are reported in Supporting Information Fig.S3.

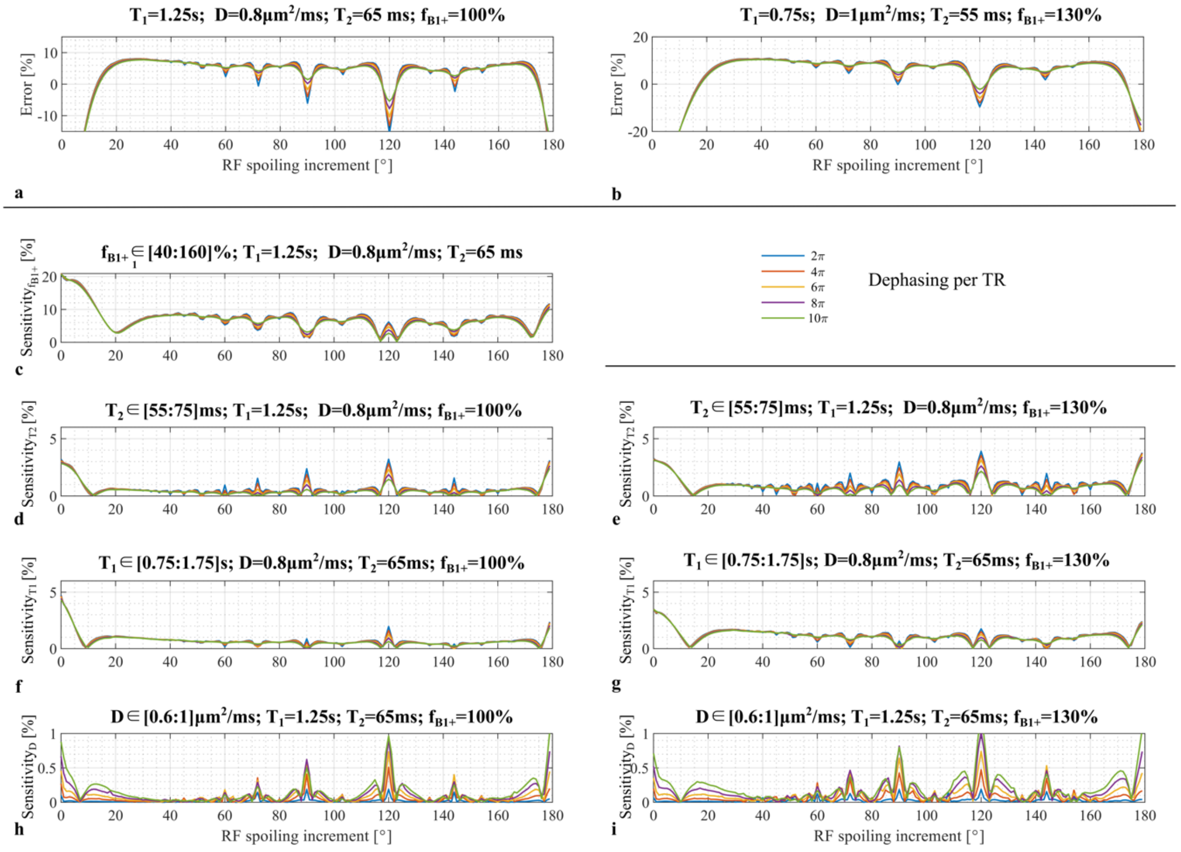

Supporting Information Figure S3: Numerical simulations, for each spoiling condition, of T_1^{app} error in two specific cases: (a) T_1=1250ms, D=0.8μm^2/ms, T_2=65ms and $f_{B_1^+}$=100%, (b) T_1=0.75s, D=1.0μm^2/ms, T_2=55ms and $f_{B_1^+}$=130%. Sensitivity of T_1^{app} to $f_{B_1^+}$ (c), the true T_2 time (d-e), the true T_1 time (f-g) and the true diffusion coefficient (h-i). The sensitivity to T_1, T_2 and D are computed in two conditions: B_1^+ efficiency of 100 % (d-f-h) or 130 % (e-g-i).

Notable differences with respect to observations at 7T are that the sensitivity of the error in T_1^{app} to T_1 and T_2 times is reduced at 3T, while the impact of the gradient-induced spoiling is larger as is the dependence of T_1^{app} on the diffusion coefficient, particularly for ϕ_0 of 72°, 90° and 120°.

Acquisitions

In vivo data were acquired in one session on one volunteer (female, 30y) with approval granted by the local ethics committee of the institution and the informed written consent of the participant. Data were acquired on a Siemens 3T Prisma scanner using the body coil for transmission and a 32 channel head coil for reception.

Multi-echo 3D SPGR data were acquired with two different flip angles, α_1=4° and α_1=25°, using an in-house sequence. The TE ranged from 2ms to 14.74ms with an echo spacing of 1.82ms. The TR was fixed to 18ms. Data were acquired over a field-of-view of 192×192×153.6mm^3 with 1.2mm isotropic resolution. The duration and amplitude of the gradients along the readout direction were identical to those simulated. Data were acquired for two spoiling conditions (net dephasing per TR of 2π or 6π across a voxel) by changing the duration of the spoiler gradient, and for five RF spoiling increments (30°, 72°, 117°, 120° and 137°). Partial Fourier (factor 6/8) was used in each phase-encoded direction to achieve a tolerable total scan time per session. Additional calibration data to map the B_1^+ efficiency were acquired using an in-house pulse sequence based on a spin-echo-stimulated-echo approach (2).

After co-registering all the weighted images together, T_1^{app} maps were constructed as described for the 7T data.

Correction factors for each protocol were computed with and without incorporating diffusion using a T_2 of 65ms and applied separately to each T_1^{app} map.

Grey and white matter masks were created as for the 7T data. Histograms of T_1 times with and without (i.e. T_1^{app}) correction in GM and WM that had a B_1^+ efficiency between 90% and 110% are plotted in Supporting Information Fig.S4 and compared with simulations of the acquisitions using T_1 times of 1s and 1.5s to approximate WM and GM respectively.

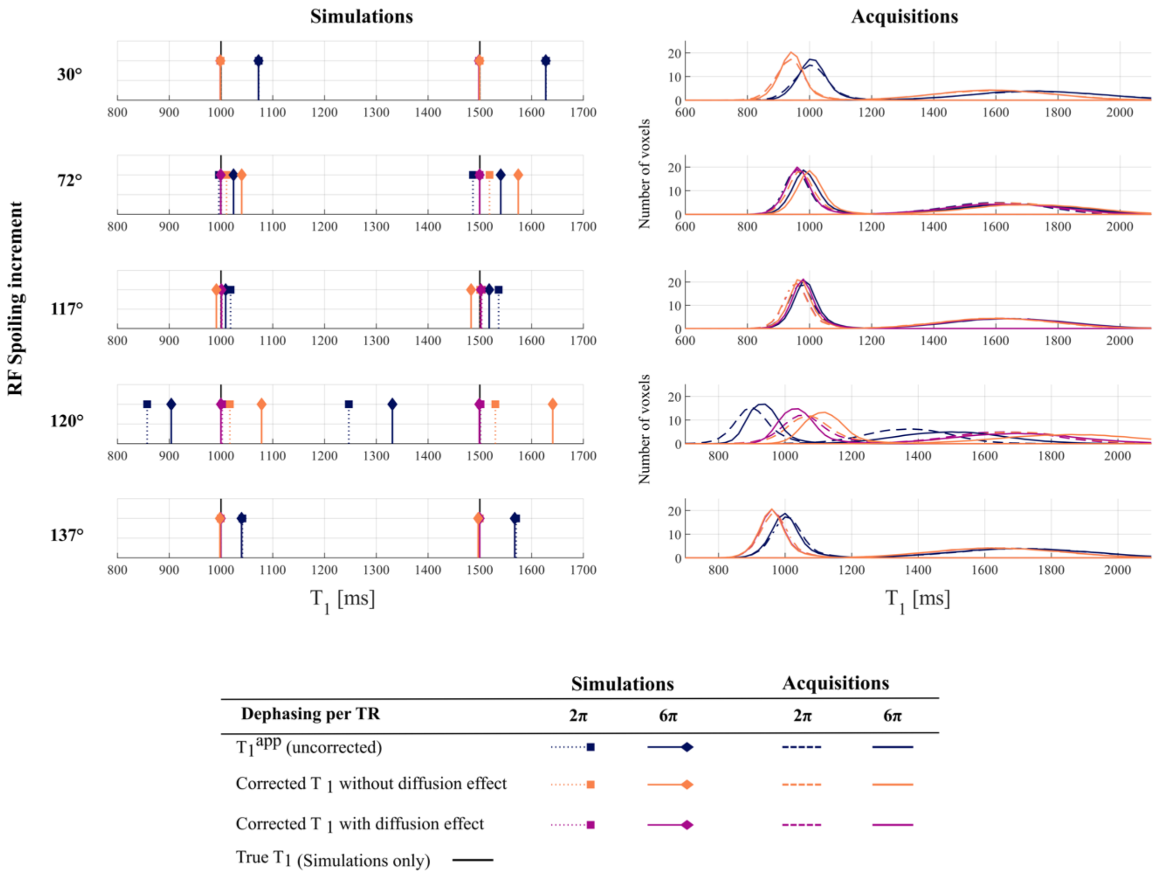

Supporting Information Figure S4: Acquisitions and numerical simulations at 3T for RF spoiling increments of 30°, 72°, 117°, 120° and 137°. T_1 before (i.e. T_1^{app}) and after correction for imperfect spoiling with correction factors determined assuming T_2 = 65ms and D = 0.8μm^2/ms or ignoring diffusion. Simulations (left): true T_1 times are indicated by a solid black line at 1s and 1.5s. *In vivo* acquisitions (right): distribution of T_1 times in GM and WM where B_1^+ efficiency was between 90% and 110%.

As at 7T, the simulations and *in vivo* experiments provided consistent results. The RF spoiling increments exhibited distinct behaviours: ϕ_0 = 30° and 137° were least dependent (coincident histograms after correction) on the gradient spoiling condition and the specifics of the correction parameters, though they also benefited most from the correction being applied; ϕ_0 = 120° was most sensitive to the gradient spoiling condition and parameters used to determine the correction factors, ϕ_0 = 72° and 117° were too but to an ever decreasing extent.

For a more comprehensive investigation of dependencies at 3T see (3).

Supporting Information 4:

The Extended Phase Graph (EPG) framework uses a Fourier approach to implement the Bloch equations underlying MR signal evolution. It not only provides a computationally efficient approach but also intuition regarding echo formation. In this formalism, magnetization is described by its occupancy of configuration states corresponding to modulated longitudinal magnetization, Z_n, and dephasing, F_n, n>0, or rephasing, F_n^*, n<0 transverse magnetization (4). n is an integer indexing the degree of dephasing ϕ, imparted by the spoiler gradient across the voxel length each TR. F_0 corresponds to the echo forming signal.

Spoiling:

Perfect spoiling would lead to magnetisation occupying only the unsaturated, unmodulated longitudinal magnetisation, Z_0, and dephasing states. Populated rephasing states can form spin echoes by refocusing through F_0, the echo-forming signal. Similarly, non-zero higher order longitudinal states can produce stimulated echoes and lead to periodic instability in the steady state signal.

Impact of diffusion**:**

Each application of the spoiler gradient imparts a specific phase patterns, e.g. 2π across a voxel, or equivalently increases the order of the configuration states, i.e. *n*. Diffusion causes a loss of coherence of these phase patterns, or equivalently a reduction of the population of the configuration state, particularly when the patterns are of high spatial frequency, i.e. higher order state in the EPG (large |n|), (5).

Application to the MPM protocols

EPG diagrams depicting the amplitude of the configuration state populations of the PD-weighted protocols used in this study are shown in Supporting Information Fig.S5. The longitudinal and rephasing transverse states are most substantially populated for ϕ_0 =120°, which as a consequence has the poorest spoiling behaviour. Occupancy of these higher order states, but with lower amplitude, is also seen for ϕ_0 =144°. Diffusion attenuates the problematic spin and stimulated echo pathways, as can be seen by contrasting the EPGs for ϕ_0 =120°, and indeed ϕ_0 =144°, with and without diffusion (c.f. columns a, c and b, d). As a result, these increments are predicted to have higher sensitivity to diffusion-related effects. This is evidenced by their greater sensitivity to the spoiler gradient moment (Fig.1, main manuscript), and to the inclusion of diffusion in calculating the correction parameters (Fig.3, main manuscript).

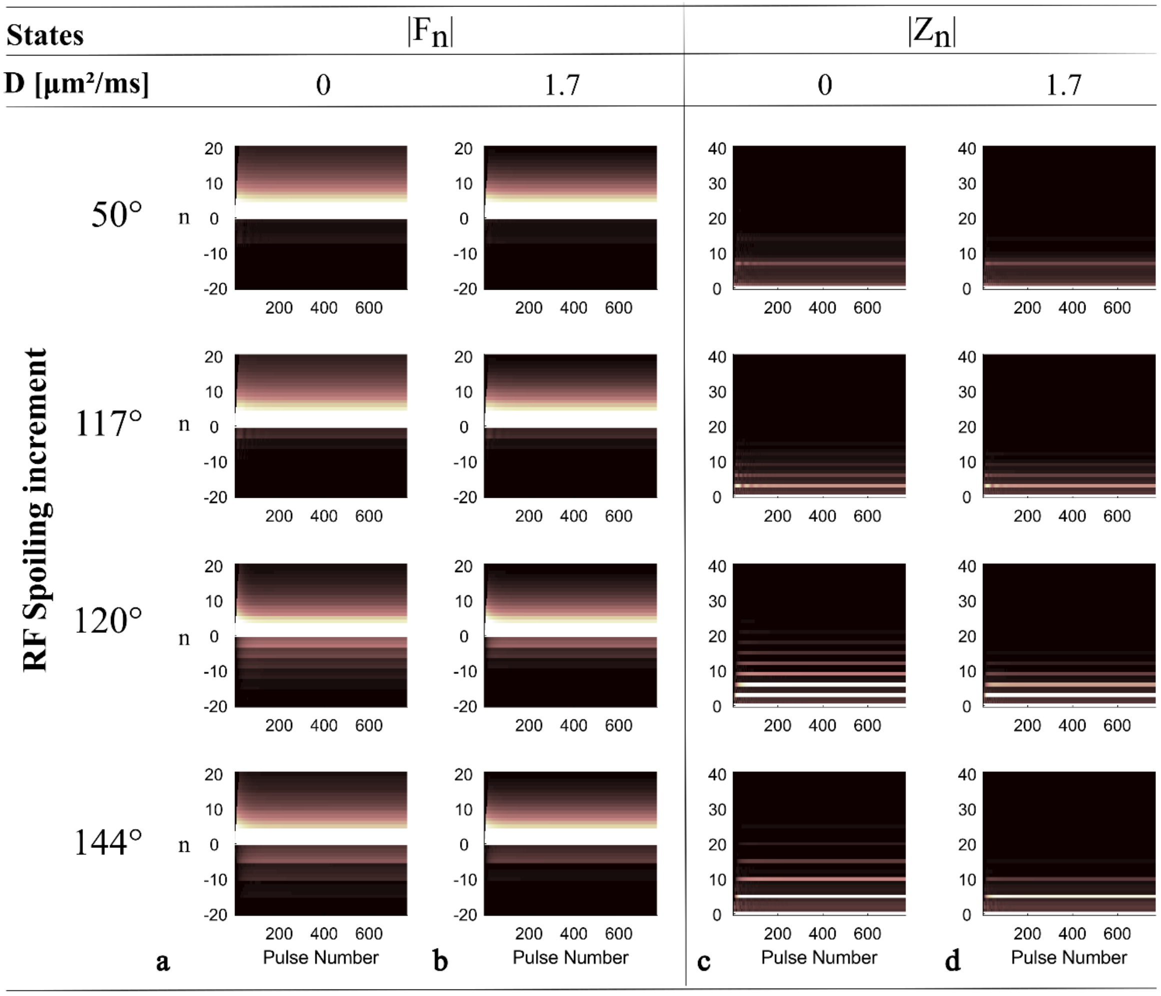

Supporting Information Figure S5: EPG diagrams, before (a, c) and after (b, d) incorporating diffusion. The diagrams depict the population amplitude of transverse, $|F_n|$ and longitudinal, $|Z_n|$, configuration states with n the degree of dephasing $\phi = 2\pi$. The simulations used a T_1 of 1000ms, T_2 of 80ms and a diffusion coefficient of 1.7μm^2/s with a flip angle of 6° and a TR of 19.5ms. Note that for visualisation purposes only a subset of the EPG diagrams are shown: $0 < n \leq 20$ for the dephasing transverse configuration states, $0 > n \geq -20$ for the rephasing transverse magnetization and $0 \leq n \leq 40$ for the longitudinal states. Higher order longitudinal and rephasing states are more populated for the RF spoiling increments of 120° and 144°. Since higher order states are especially attenuated by diffusion, incorporating this effect has the most appreciable impact on these increments. It can also be seen that sufficient pulses are incorporated to reach a steady state, which is arrived at comparatively quickly for all increments.

The populations of the problematic rephasing states depend not only on diffusion but on the flip angle, or equivalently the B_1^+ efficiency, and on how quickly the magnetisation itself decays, i.e. the T_2 time. The intra-voxel magnetisation distributions and the first six population amplitudes of the rephasing configuration states are shown in Supporting Information Fig.S6 for two flip angles, corresponding to the PDw and T_1w protocols used in this study, and two T_2 times. For the increments of 120° and 144°,

the rephasing states of both the PDw and T1w signals had appreciable amplitudes, which were modulated by the T_2 time. For the 50° and 117° increments, the spoiling was efficient for the low flip angle of the PDw acquisition, as evidenced by lower population amplitudes for the rephasing states regardless of T_2 time (Supporting Information Fig.S6b, blue and orange, particularly for 50°). However, at higher flip angle the spoiling efficiency was reduced, leading to larger population amplitudes in the rephasing states for the T_1w acquisition (Supporting Information Fig.S6b yellow and purple). With $\phi_0 = 50°$ the occupancy of these states was particularly sensitive to the flip angle, and therefore the B_1^+ efficiency, causing T_1^{app} computed from this RF spoiling increment to be heavily dependent on $f_{B_1^+}$ (Fig.1,3,4 of the main manuscript), and by extension T_2.

Such an interaction between flip angle and T_2 sensitivity leads to an apparent $f_{B_1^+}$ dependence that is in fact T_2-driven and makes the increment sensitive to the T_2 time used to compute the post-hoc correction factors (Figs.2,7 of the main manuscript). The lack of such an interaction for the 144° increment results in T_1^{app} being comparatively T_2 insensitive (Figs.1,3,5 of the main manuscript) and likely underpins its robustness to the choice of T_2 for the computation of post-hoc correction factors (Figs.2,7 of the main manuscript).

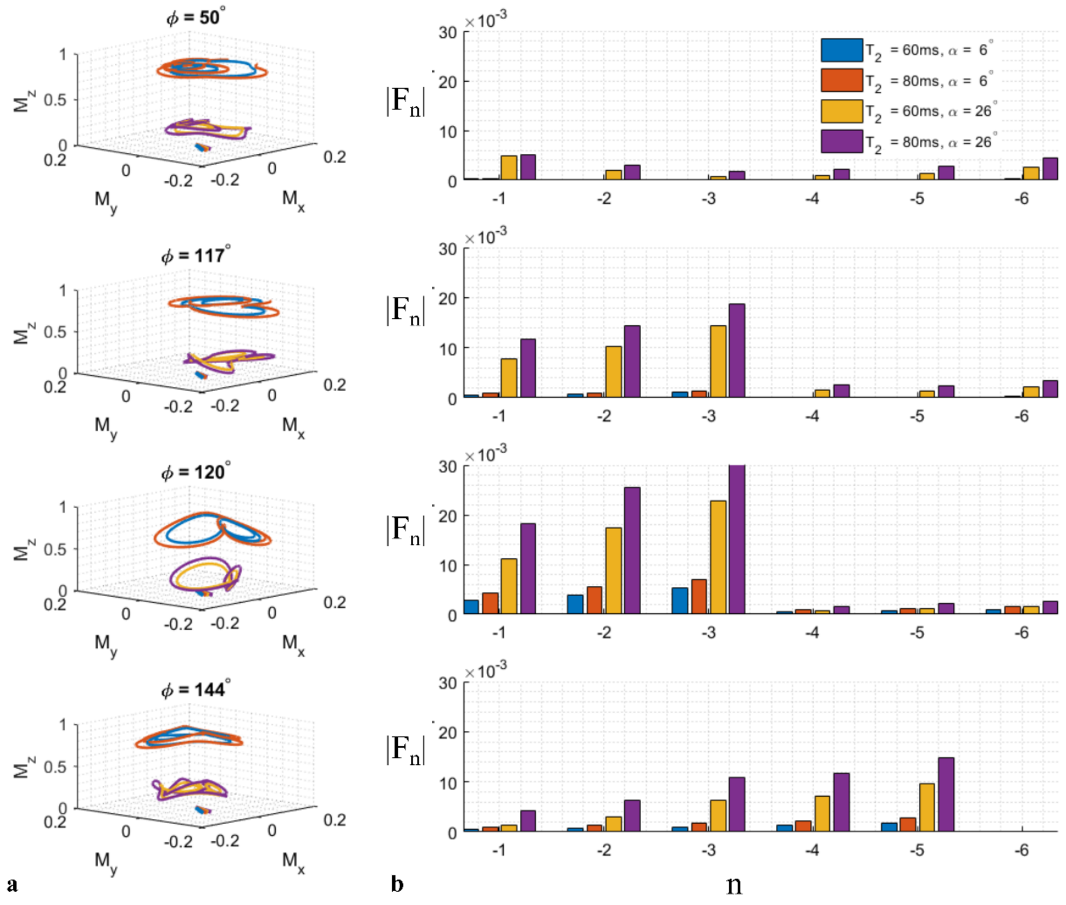

Supporting Information Figure S6: Intra-voxel magnetisation distribution derived from the steady-state EPG coefficients for each increment (a), and the population amplitudes of the first six rephasing transverse configuration states (b). Four cases are shown: two nominal flip angles (6° and 26° corresponding to the PDw and T1w acquisitions of this study) and two T_2 times (60ms and 80ms). All other simulation settings are as in Supporting Information Fig.S5. The net SPGR echo-forming signal (i.e. F_0 state) is projected onto the transverse plane in (a) with an artificial phase dispersion added to aid visualisation.

References

1. Dathe H, Helms G. Exact algebraization of the signal equation of spoiled gradient echo MRI. Phys. Med. Biol. 2010;55:4231–4245 doi: 10.1088/0031-9155/55/15/003.

2. Lutti A, Hutton C, Finsterbusch J, Helms G, Weiskopf N. Optimization and Validation of Methods for Mapping of the Radiofrequency Transmit Field at 3T. Magn Reson Med 2010;64:229–238 doi: 10.1002/mrm.22421.

3. Corbin N, Malik SJ, Callaghan MF. The in vivo impact of diffusion spoiling on the estimate of T1 using spoiled gradient echoes with variable flip angles. In: ISMRM, Montreal. ; 2019. p. 7507.

4. Scheffler K. A pictorial description of steady-states in rapid magnetic resonance imaging. Concepts in Magnetic Resonance 1999;11:291–304 doi: https://doi.org/10.1002/(SICI)1099-0534(1999)11:5<291::AID-CMR2>3.0.CO;2-J.

5. Weigel M, Schwenk S, Kiselev VG, Scheffler K, Hennig J. Extended phase graphs with anisotropic diffusion. Journal of Magnetic Resonance 2010;205:276–285 doi: 10.1016/j.jmr.2010.05.011.
